# Interactions of fentanyl with blood platelets and plasma proteins: platelet sensitivity to prasugrel metabolite is not affected by fentanyl under in vitro conditions

**DOI:** 10.1007/s43440-023-00447-7

**Published:** 2023-01-17

**Authors:** Radosław Bednarek, Boguslawa Luzak, Jacek Golański, Magdalena Boncler

**Affiliations:** 1 Department of Cytobiology and Proteomics, Chair of Biomedical Sciences, Medical University of Lodz, Ul. Mazowiecka 6/8, 92-215 Lodz, Poland; 2 Department of Haemostasis and Haemostatic Disorders, Chair of Biomedical Sciences, Medical University of Lodz, Ul. Mazowiecka 6/8, 92-215 Lodz, Poland

**Keywords:** Fentanyl, Blood platelets, Platelet activation, Antiplatelet therapy, Drug carriers, Protein binding

## Abstract

**Background:**

Clinical trials indicate that fentanyl, like morphine, may impair intestinal absorption and thus decrease the efficacy of oral P2Y_12_ inhibitors, such as clopidogrel, ticagrelor, and prasugrel. However, the ability of fentanyl to directly negate or reduce the inhibitory effect of P2Y_12_ receptor antagonists on platelet function has not been established. A series of in vitro experiments was performed to investigate the ability of fentanyl to activate platelets, potentiate platelet response to ADP, and/or diminish platelet sensitivity to prasugrel metabolite (R-138727) in agonist-stimulated platelets. The selectivity and specificity of fentanyl toward major carrier proteins has been also studied.

**Methods:**

Blood was obtained from healthy volunteers (19 women and 12 men; mean age 40 ± 13 years). Platelet function was measured in whole blood, platelet-rich plasma and in suspensions of isolated platelets by flow cytometry, impedance and optical aggregometry. Surface plasmon resonance and molecular docking were employed to determine the binding kinetics of fentanyl to human albumin, α_1_-acid glycoprotein, apolipoprotein A-1 and apolipoprotein B-100.

**Results:**

When applied at therapeutic and supratherapeutic concentrations under various experimental conditions, fentanyl had no potential to stimulate platelet activation and aggregation, or potentiate platelet response to ADP, nor did it affect platelet susceptibility to prasugrel metabolite in ADP-stimulated platelets. In addition, fentanyl was found to interact with all the examined carrier proteins with dissociation constants in the order of 10^–4^ to 10^–9^ M.

**Conclusions:**

It does not seem that the delayed platelet responsiveness to oral P2Y_12_ inhibitors, such as prasugrel, in patients undergoing percutaneous coronary intervention, results from direct interactions between fentanyl and blood platelets. Apolipoproteins, similarly to albumin and α_1_-acid glycoprotein, appear to be important carriers of fentanyl in blood.

**Supplementary Information:**

The online version contains supplementary material available at 10.1007/s43440-023-00447-7.

## Introduction

Fentanyl is a drug from the opioid family that is used for surgical anesthesia and for analgesia in the treatment of chronic pain [1]. Its strong analgesic and anesthetic effects are exerted primarily by the activation of μ-opioid receptors, which are widely expressed in the central nervous system. The μ-opioid receptors are also abundantly distributed in the enteric nervous system, where they regulate gastrointestinal tract motility in the secretion or transport of fluids and electrolytes [2]. Furthermore, these opioid receptors have been identified in immune cells, indicating that the opioid system plays a role in regulating intestinal inflammation and intestinal ischemia [3].

Nearly 40 years ago, Mehrishi et al. demonstrated the binding of naloxone (an opioid antagonist) to blood platelets, thus suggesting that platelets express functional μ-opioid receptors [4]. Until now, the importance of opioid receptors in platelet functioning remains unclear, since no other reports identifying opioid receptors on platelets in humans have been published. Interestingly, μ-, δ-, and κ-opioid receptors have been recently detected in mouse platelets [5]. In addition, several clinical trials (PERSEUS, PACIFY, ON-TIME 3) have consistently demonstrated that fentanyl attenuates the antiplatelet effects of the P2Y_12_ inhibitor ticagrelor in patients undergoing percutaneous coronary intervention (PCI) [6–9]. However, the mechanism by which fentanyl could interact with antiplatelet drugs, and thus impair their effectiveness in antiplatelet therapy, is obscure. It is likely that this effect is related to the potential of fentanyl to reduce gastrointestinal motility and delay gastric emptying, which reduce and delay absorption of oral platelet inhibitors [10, 11]. It is also possible that fentanyl could directly activate platelets via μ-opioid receptors and thus antagonize the effects of antiplatelet drugs. To date, no data exists regarding the effect of P2Y_12_ inhibitor on platelet function in vitro in the presence of fentanyl.

The ability of fentanyl to regulate platelet function may strongly depend on the presence of plasma proteins in the cellular environment, as fentanyl is extensively bound by plasma proteins [12]. The first report on the interactions of fentanyl with blood components date back to the 1970s, when it was evidenced that fentanyl can bind to plasma proteins, such as albumin [13]. Since then, fentanyl has been demonstrated to bind to lipoproteins [14], erythrocytes [15], membrane oxygenator used for gas exchange during cardiopulmonary bypass [16], ultrafiltration devices and plasticware [12]. The binding of fentanyl to fibrinogen has been excluded as the percent of fentanyl bound to plasma and serum was found to be similar [14].

When analyzing literature, it is worth paying attention to the analytical tools used to estimate the binding ability of fentanyl to macromolecules. In most cases, separative methods were applied to investigate fentanyl–protein binding (i.e., equilibrium dialysis, ultrafiltration); as such, most data are restricted to the information on the fentanyl fraction bound to a protein in percent of the total drug concentration. However, little is known on the binding sites of fentanyl on plasma proteins, the strength of these interactions, and the extent to which these interactions might occur (affinity and kinetic rate constants).

The present study fills the gap regarding our understanding of the mechanisms by which fentanyl impairs the efficacy of oral P2Y_12_ inhibitors and extends existing knowledge about the interaction of fentanyl with plasma proteins. We hypothesize that, as the analgesic effect of fentanyl (and other μ-opiate receptor agonists) is associated with activation of μ-opioid receptors and G_i_ protein-dependent inhibition of cyclic AMP (cAMP) production [17], fentanyl may directly affect platelet function mainly via μ-opioid receptors, in a similar way to morphine [4, 18, 19]. More specifically, we suppose that fentanyl activates the G_i_ protein via μ-opioid receptors, leading to the inhibition of adenylate cyclase, and subsequently the reduction of cAMP level and platelet activation. Accordingly, this G_i_ protein-activating effect of fentanyl could abrogate the effects of P2Y_12_ antagonists, which inhibit platelet activation through the P2Y_12_/G_i_-dependent mechanism. The study assesses the influence of fentanyl on platelet function under different experimental conditions, more specifically, its ability to stimulate platelet function, potentiate the platelet response to ADP, and/or diminish platelet sensitivity to prasugrel metabolite. It also examines the selectivity and specificity of fentanyl toward major carrier proteins, including apolipoproteins.

## Materials and methods

### Chemicals

Fentanyl citrate (N-phenyl-N-[1-(2-phenylethyl)-4-piperidinyl]propanamide citrate) was purchased from Toronto Research Chemicals (Toronto, Canada). Prasugrel metabolite (R-138727) was obtained from BOC Sciences (Shirley, NY, USA). The CM5 sensor chips, the Amine Coupling Kit, CNBr-activated Sepharose and HBS-EP buffer were from Cytiva (Global Life Sciences Solutions Poland, Warsaw, Poland). Human serum albumin (HSA), bovine serum albumin (BSA), α_1_-acid glycoprotein (α_1_-AGP) from human plasma, apolipoprotein A-1 (ApoA-1) from human plasma, apolipoprotein B-100 (ApoB-100) from human plasma, adenosine 5′-diphosphate (ADP), prostaglandin E_1_ (PGE_1_) and adrenaline were obtained from Sigma (St. Louis, MO, USA). Monoclonal antibodies (anti-human CD61/PerCP, CD62/PE, mouse IgG1/PE isotype control), Cellfix and blood collection tubes containing 3.2% buffered sodium citrate solution were obtained from Becton Dickinson (Franklin Lakes, New Jersey, USA). S-Monovette® neutral tubes were purchased from Sarstedt (Nümbrecht, Germany). Glass cuvettes and stirring bars were purchased from Chrono-Log (Havertown, PA, USA). Sterile saline solution (0.9%) was purchased from Glenmark Pharmaceuticals (Warsaw, Poland). All other chemicals, unless otherwise stated, were purchased from Avantor Performance Materials (Gliwice, Poland). Water used for solution preparation and glassware washing was passed through an Easy Pure UF water purification system (Barnstead Thermolyne, Dubuque, IA, USA).

### Preparation of compound solutions

Stock solutions of fentanyl, prasugrel metabolite, ADP and adrenaline were made in saline or phosphate-buffered saline (PBS) and stored at ‒ 80 °C until use. Working solutions of these compounds were freshly prepared by dilution of the stock solutions with saline or PBS buffer, pH 7.4, at concentrations 50-fold higher than the intended final concentrations.

### Participants

Human blood was obtained from consecutively recruited healthy volunteers: 19 women and 12 men; mean age 40 ± 13 years. All individuals provided their written informed consent to participate in the study. All individuals confirmed not having taken medications known to influence platelet function (e.g., non-steroidal anti-inflammatory drugs, NSAIDs) for at least 2 weeks prior to the study. The study was approved by the Human Studies Committee of the Medical University of Lodz (RNN/153/20/KE, June 16, 2020) and was conducted in accordance with the guidelines established by the Declaration of Helsinki. The study was carried out after gaining permission from the Regional Pharmaceutical Inspectorate in Lodz to conduct research using fentanyl.

### Blood collection

The blood samples taken for the measurement of platelet function were collected according to the guidelines for performance of multi-color flow cytometry in whole blood [20] and the ISTH recommendations for the standardization of light transmission aggregometry [21]. Briefly, blood samples were drawn from fasting participants in the morning between 8.00 and 9.00 a.m. with light tourniquets and short and moderate phlebostasis from the antecubital vein using a 21-gauge needle. Blood was collected in 9 ml polypropylene tubes (S-Monovette®) prefilled with a 3.2% sodium citrate solution with the blood-to-anticoagulant ratio of 9:1 vol/vol.

### Processing of blood samples

Functional platelet tests were performed in whole blood, platelet-rich plasma (PRP) and suspensions of isolated platelets. Platelet-rich plasma was prepared by 12 min of 200×*g* centrifugation of a withdrawn blood at 37 °C. Platelet-poor plasma (PPP) was obtained by centrifuging PRP samples at 2000×*g* for 15 min at 37 °C. Platelets were isolated from PRP by gel-filtration on albumin-coupled Sepharose 4B columns. The columns were equilibrated and eluted with Tyrode buffer (134 mM NaCl, 12 mM NaHCO_3_, 2.9 mM KCl, 0.34 mM Na_2_HPO_4_, 1 mM MgCl_2_, 10 mM HEPES, 5 mM glucose, pH 7.4). Platelet count in PRP and gel-filtered platelets was measured with a Sysmex XS-800i™ automated hematology analyzer (Sysmex, Kobe, Japan). The mean final platelet count was 2.4 ± 0.3 × 10^8^ cells per ml in PRP and 1.6 ± 0.3 × 10^8^ cells per ml in gel-filtered platelets.

### Studies of fentanyl on platelet function

The effect of fentanyl on platelet function was studied at therapeutic (2 ng/ml, equivalent to 3.8 nM) and non-therapeutic (2000 ng/ml, equivalent to 3.8 µM) concentrations, regardless of the method applied. The recommended effective fentanyl concentration in plasma is 1–2 ng/ml for analgesia and 10–20 ng/ml for anesthesia [22, 23]. The concentrations of fentanyl and other opioids used in in vitro studies range between nanomolar and micromolar concentrations [24]. Each experiment examining the effect of fentanyl on platelet function (studies of platelet activation and aggregation) consisted of three parts. The first examined the effect of fentanyl alone, which was added to the samples instead of agonist. The second studied the influence of fentanyl on platelet function in the presence of agonist, evaluating the ability of fentanyl to enhance agonist-induced platelet function: in this in vitro model, the biological material was incubated with fentanyl for 15 min at 37 ℃ before a test, or fentanyl was added to the biological material together with the agonist. Finally, the third part examined the effect of fentanyl on agonist-induced platelet function in the presence of prasugrel metabolite (PM) to determine whether fentanyl was able to abolish the inhibitory effects of prasugrel metabolite on platelet function; briefly, before each measurement, the biological material was preincubated with fentanyl and PM for 15 min at 37 ℃. As micromolar PM concentrations have been shown to inhibit platelet aggregation in whole blood [25], the inhibitory effects of PM on platelet function were assessed at the concentrations of 1.3 or 2 µM.

The ability of fentanyl to enhance agonist-induced platelet aggregation in whole blood or PRP was examined in the presence of subthreshold ADP concentration (2 µM). The exceptions were experiments with prasugrel metabolite, where ADP was applied at 5 µM, in studies of platelet aggregation measured under constant stirring conditions, or at 10 µM, in experiments of platelet activation measured under static conditions. The potential effects of fentanyl on agonist-induced platelet activation were measured in whole blood, PRP and suspensions of isolated platelets after 5-min stimulation with 2 µM ADP (in the model without PM) or 10 µM ADP (in the model with PM).

The action of fentanyl on blood platelets was verified by a number of control experiments, in which saline and ADP or ADP in combination with adrenaline (10 µM) were used instead of fentanyl, depending on the model applied. Adrenaline was used as a positive control in experiments with fentanyl and ADP, as it has ability to potentiate platelet response to agonists [26, 27]. The concentrations of agonists in platelet functional tests were selected based on previous reports [26, 28] and our own data.

#### Impedance aggregometry

Impedance aggregometry was used to assess the effect of fentanyl on platelet aggregation in whole blood. The measurement procedure was performed in accordance with the manufacturer’s instructions. Briefly, whole blood (300 µl) was mixed with 0.9% saline (300 µl) and prewarmed to 37 °C, in the test cell for 3 min. Then, depending on the model, fentanyl alone or agonist with/without fentanyl were added and platelet aggregation was recorded continuously for 6 min. using a Multiplate^®^ analyzer (Roche Diagnostics GmbH, Mannheim, Germany). The area under the aggregation curve (AUC), known as preferred output parameter for the interpretation of platelet aggregation in whole blood, was used to express the aggregation response over the measured time, as described previously [29]. All measurements were completed within 3 h after blood collection.

#### Light transmission aggregometry

In the models described above, light transmission aggregometry (LTA) was used to evaluate platelet aggregation in platelet-rich plasma treated with fentanyl. Using this method, the study also examined the potentiation of platelet response by fentanyl in the two additional models. To determine whether fentanyl has ability to enhance platelet aggregation induced by the agonist at ADP concentrations other than subthreshold, platelet aggregation was investigated in the presence of fentanyl (2 µg/ml) at a broad range of ADP concentrations (0.1‒20 µM). In addition, to test whether fentanyl is able to reduce or abolish platelet response to prostaglandin E_1_ (PGE_1_), which is known to stimulate cAMP synthesis and inhibit platelet aggregation, the platelet response to 5 µM ADP in the presence of 2 µg/ml fentanyl was measured after one-minute preincubation with 0.1 µM PGE_1_.

Platelet aggregation was monitored for 10 min on a dual-channel aggregometer (Chrono-Log, Havertown, PA, USA) according to the guidelines of the SSC/ISTH [21]. Maximum aggregation (*A*_max_) and AUC were the readout parameters included in the analysis. Each experiment was completed within a maximum of four hours after blood sampling.

### Analysis of platelet activation

The effect of fentanyl on platelet activation was assessed in whole blood, PRP or suspensions of isolated platelets by flow cytometry using the anti-human CD61/PerCP and anti-human CD62/PE antibodies. Non-specific binding was assessed using mouse IgG1/PE isotype control. In blood samples, platelets were distinguished from other blood cells on the basis of the binding of anti-CD61 antibodies, whereas the measurements in PRP and suspensions of isolated platelets were performed with the whole cell population without gating. Platelet response, i.e., the fraction of CD62P-positive objects, was measured after platelet incubation with fentanyl in the models described above. The cells were stained with antibodies prior to fixation (blood) or after fixation with 1% paraformaldehyde (PRP, suspensions of isolated platelets), as described previously [30, 31]. This approach had become necessary due to the large number of samples processed for each experiment. The fractions of CD62P-positive platelets were determined with a FACSCanto II cytometer (Becton–Dickinson, Franklin Lakes, NJ, USA). Ten thousand events (CD61/PerCP-positive objects) per sample were acquired and data were processed with FACS/Diva software (Becton–Dickinson, Franklin Lakes, NJ, USA).

### Surface plasmon resonance spectroscopy

Surface plasmon resonance (SPR) is a non-separative, moderate-throughput laboratory technique successfully used to rank drug molecules of weak, medium, and strong affinity to carrier proteins from human plasma. Herein, the SPR technique was used to study the kinetics of interactions between fentanyl or prasugrel metabolite and human plasma proteins, including human serum albumin (HSA), α_1_-acid glycoprotein (α_1_-AGP), apolipoprotein A-1 (ApoA-1), and apolipoprotein B-100 (ApoB-100) with the Biacore X system (Biacore AG, Uppsala, Sweden). The ligand (plasma protein) was immobilized on the CM5 sensor chip, whereas the analyte (solution of fentanyl or PM) was passed over the surface of the chip, as described below.

Human albumin, α_1_-AGP, ApoA-1 and ApoB-100 solutions were diluted to a concentration of 100 µg/ml in 10 mM sodium acetate buffer, pH 4.0. The diluted protein samples were injected at a volume of 50 μl and at a flow rate of 5 µl/min (contact time: 600 s) and then covalently attached to the surface of a CM5 sensor using amine coupling chemistry, according to the manufacturer’s instructions. The response was expressed in resonance units (RU), an arbitrary unit specific for the Biacore instrument (1000 RU corresponds to ∼1 ng of bound protein per mm^2^). The ranges of SPR signal after binding of the examined proteins to CM5 sensor were as follows: 5900 RU for HSA, 1450 RU for α_1_-AGP, 1900 RU for ApoA-1, and 1700 RU for ApoB-100. Plasma proteins were coupled to the Fc1 flow cells of each sensor. Concurrently, Fc2 flow cells were subjected to amine coupling procedure without any protein (sodium acetate buffer with no protein was applied during sample injection). Subsequently, Fc2 flow cells were used as controls for assessing binding specificity and as blank samples for subtraction of the bulk refractive index background. The degree of binding by 10, 20, 40, and 80 µM fentanyl, or 2, 5 and 10 µM prasugrel metabolite, was measured over immobilized plasma proteins at 25 °C. HBS-EP buffer (0.01 M HEPES, pH 7.4, 0.15 M NaCl, 3 mM EDTA, 0.005% Surfactant P20) was used as a running buffer with a flow rate of 30 µl/min and injection volume of 50 µl (contact time: 100 s). This was followed by 100 s dissociation time. Regeneration of the surfaces was achieved by injection of 20 µl 10 mM glycine–HCl, pH 3.0, (contact time: 40 s.). Sensorgrams were analyzed using BIAevaluation 4.1.1 software supplied by Biacore AB (Uppsala, Sweden). The association rate constant (k_a_), and dissociation rate constant (k_d_), were determined from individual association and dissociation phases, respectively, by global fitting of the data using the Langmuir binding model with drifting baseline, assuming one-to-one interactions superimposed on a linear baseline drift. Each dissociation constant (K_D_) was obtained by calculating the ratio of the dissociation and association rate constants: k_d_/k_a_ = K_A_ = K_D_^−1^.

### Molecular docking

Molecular docking of fentanyl to plasma carrier proteins was performed with the AutoDock 4.2.6 software implemented in AutoDockTools 1.5.7 suite (The Scripps Research Institute, La Jolla, CA, USA) [32], using the Lamarckian genetic algorithm (GA) for a flexible ligand and a rigid receptor docking. The in silico analysis was carried for the proteins with the X-ray structure available in a public data base, i.e., HSA, α_1_-AGP, and ApoA-1.

The 3D conformer of fentanyl (CID: 3345; IUPAC name: N-phenyl-N-[1-(2-phenylethyl)piperidin-4-yl]propanamide) was downloaded from PubChem (http://pubchem.ncbi.nlm.nih.gov) in SDF file format and converted to PDB file format using Open Babel 2.4.1 software [33]. The ligand molecules were prepared for the proper use of AutoDock semi-empirical free energy force field as follows: hydrogen atoms were added, Gasteiger charges were computed, 28 non-polar hydrogens were merged, 12 aromatic carbons were detected, the torsional degrees of freedom (TORSDOF) were set to six, and six rotatable (flexible) and 26 rigid bonds were identified. The coordinates for the center of the ligand molecule were calculated by the UCSF Chimera 1.16 software using the “Define Centroid” tool. The resulting centroid coordinates, that were subsequently used for all docking simulations, were as follows: *x* =  − 0.087, *y* =  − 0.061, *z* =  − 0.013. For the analysis, the van der Waals radii scaling factor was set to 1.00.

The three X-ray high resolution crystal structures of HSA, three X-ray high resolution crystal structures of α_1_-AGP, as well as three X-ray high resolution crystal structures of ApoA-1, were obtained from the Research Collaboratory for Structural Bioinformatics Protein Data Bank (RCSB PDB; http://www.rcsb.org) and subsequently used to predict the binding site of fentanyl with a given protein. The following crystal structures of HSA were used to verify the interactions with fentanyl: 2BXD, 2BXG, and 2VUE. The 2BXD structure is complexed with warfarin (R-enantiomer) and contains a major binding pocket for drugs (Sudlow site I) located in the core of subdomain IIA [34]. This binding site comprises six helices of the subdomain IIA (residues 196‒297) and a loop-helix feature of the subdomain IB (residues 148–154). The following Sudlow site I residues were taken into consideration for molecular docking study: TYR150, GLU153, LYS195, GLN196, LYS199, PHE211, TRP214, ALA215, ARG218, ARG222, LEU238, HIS242, ARG257, ILE264, HIS288, ALA291. The 2BXG structure complexes with ibuprofen and contains a second drug binding site (Sudlow site II), which consists of six helices located in subdomain IIIA (residues 384–497). The following Sudlow site II residues were considered during the study: ARG348, LYS351, GLU383, LEU387, ARG410, TYR411, LYS414, LEU430, VAL433, GLU450, LEU453, SER480, LEU481, VAL482, ARG485, SER489. The 2VUE structure is complexed with 4Z,15E-Bilirubin-IXα (Biliverdine-IXα) and contains the drug site III within IB subdomain, covering the following amino acid residues: LEU115, VAL116, ARG117, PRO118, GLU119, MET123, PHE134, TYR138, GLU141, ILE142, HIS146, PHE149, TYR150, LEU154, PHE157, TYR161, PHE165, LEU182, ARG186, GLY189, LYS190, LYS195. The following PDB structures of α_1_-AGP were used to verify the interactions with fentanyl: 3APU, 3KQ0, 7OUB. The 3APU structure is the genetic variant termed as α_1_-AGP1 (UniprotKB: P02763) and contains two α_1_-AGP polypeptide chains: chain A and chain B complexed with two chains of tetraethylene glycol. The 3KQ0 entry represents the genetic variant α_1_-AGP2 (UniprotKB: P19652) and is complexed with (2R)-2,3-dihydroxypropyl acetate, and the chloride ion Cl^−^. The structure 7OUB represents the genetic variant α_1_-AGP2 (UniprotKB: P19652) and contains 7-hydroxystaurosporine as the co-crystalized ligands. The following PDB structures of ApoA-1 were used to verify the interactions with fentanyl: 1AV1, 2N5E, 3R2P. The ApoA-1 structures do not contain any co-crystalized ligands. When a structure contained more than one polypeptide chain, the chain of interest was chosen for molecular docking simulations due to the best fit to the electron density, based on the data from the Full wwPDB X-ray Structure Validation Report documents available for all PDB structures. In virtually all the protein structures, all possible histidine tautomers with the proton on either the Nδ1 or Nε2 atoms were sampled; however, only the HIS242 residue found in Sudlow site I was sampled as Nδ1 protonated tautomer in 2BXD HSA structure. The co-crystalized ligands, additional polypeptide chains and water molecules were removed from the plasma protein structures by UCSF Chimera 1.16. Using the three different crystal structures of HSA, fentanyl was docked into the three abovementioned HSA drug-binding regions defined by the grid box tool of the AutoDockTools software.

Molecular docking simulations were performed at two stages to investigate the interactions of fentanyl with α_1_-AGP and ApoA-1. In the first, blind docking was performed. For this purpose the grid boxes were set to cover the whole crystal structures of plasma proteins. In this way, the fentanyl binding sites were preliminarily identified within the plasma protein structures. These preliminary sites then served to define the narrower, more specific grid boxes for the second stage of docking, termed herein as site-specific docking. The spacing for blind docking grid boxes was set to 1.000 Å, whereas the spacing for site-specific docking grid boxes was set to 0.375 Å. The detailed parameters of the grid boxes, including their sizes with the grid box center coordinates, are shown in Supplementary Materials.

Docking was performed using Lamarckian genetic algorithm (GA) with the following parameters: the maximum number of energy evolutions was 5,000,000, GA population size was 150, maximum number of generations was 27,000, the number of top individuals to survive to the next generation was 1, and the number of GA runs was 60. The protein–ligand complex with the lowest Gibbs free binding energy (ΔG_b_) and inhibition constant (K_i_) values was taken as the most optimal conformation and used for subsequent analysis.

### Statistical analysis

The results are expressed as arithmetic mean ± SD unless otherwise stated. Two, three or four groups were used for comparison, depending on the experimental model and assay applied. Differences between two groups were assessed with paired *t* test or Wilcoxon's signed rank, depending on whether the collected data met the assumption of normality. The extra sum-of-squares F-test was used to define whether significant differences existed between the dose–response curves. The EC_50_ values were calculated from dose–response plots using non-linear regression analysis. When the number of groups was > 2, data were compared by ANOVA tests: repeated measures ANOVA (RM ANOVA) or Friedman’s test. When a significant effect was observed, post hoc analyses were performed using the Dunnett’s (experiments without prasugrel metabolite or PGE_1_), Tukey’s (experiments with prasugrel metabolite or PGE_1_), or Dunn’s multiple comparisons tests (data that failed to meet assumptions for parametric analysis). Data were checked for normality, equal variance and sphericity. The Shapiro–Wilk test and Levene’s test were used to confirm that the data was normally distributed and homogenous. Non-normal distributions were transformed to logarithms for statistical analysis. Sphericity was verified with Mauchley’s test. The Geisser‒Greenhouse (G‒G) correction was applied when the sphericity assumption was not met. The statistical analysis was performed using the following software packages: Statistica v.13.3, GraphPad Prism v.5.03 or v.8.0.1, and StatsDirect v.3.3.4.

## Results

### Effect of fentanyl on platelet aggregation in whole blood

**Fig. 1:**
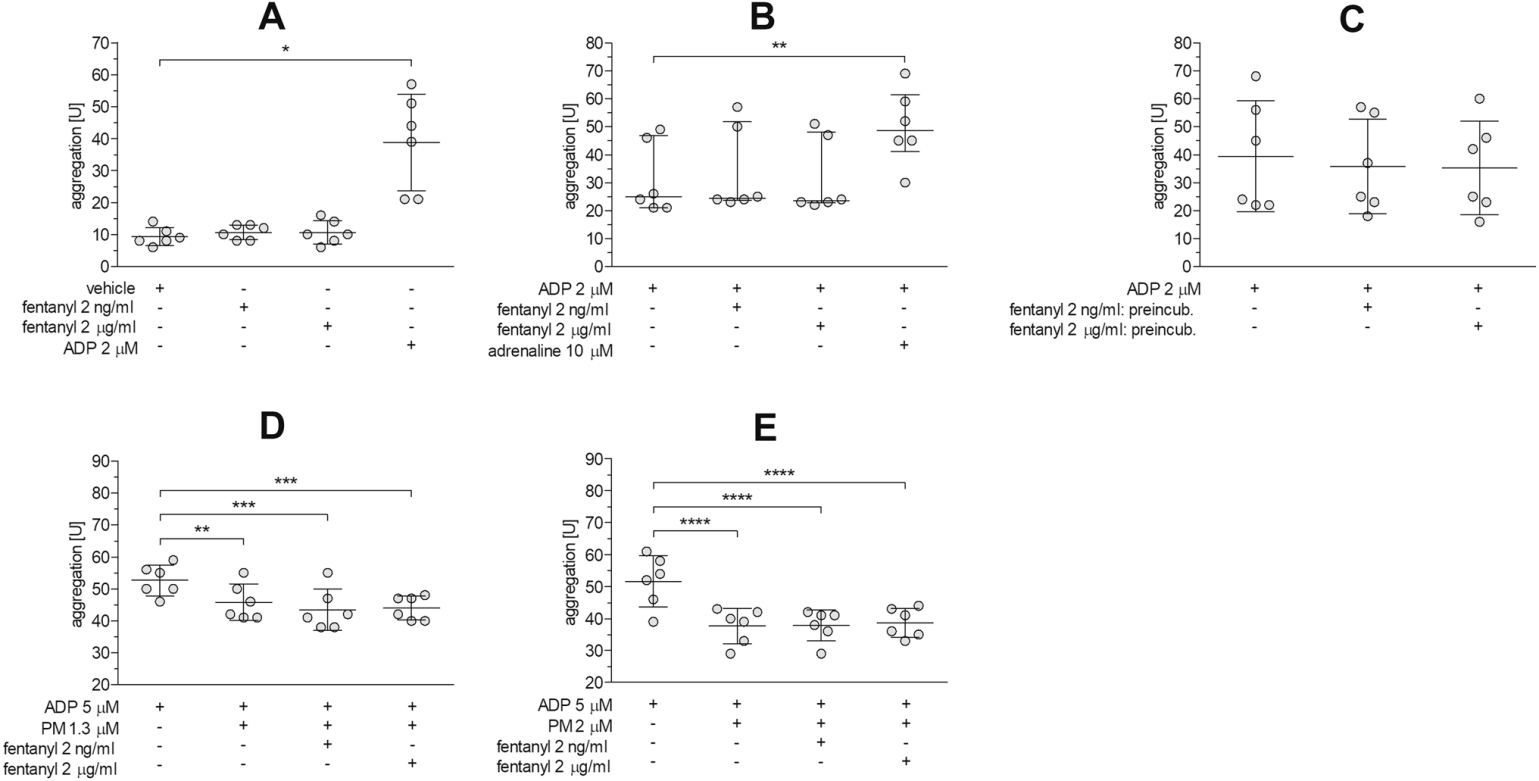
Effect of fentanyl on platelet aggregation in whole blood. The influence of 2 or 2000 ng/ml fentanyl on whole blood platelet aggregation was measured in non-stimulated cells (**A**), cells stimulated with 2 µM ADP in combination with fentanyl (**B**, **C**), and cells stimulated with 5 µM ADP after 15-min preincubation with prasugrel metabolite and fentanyl (**D**, **E**). Data with normal distribution (**A**, **C**‒**E**) are expressed as mean ± SD, and those with non-normal distribution (**B**) are given as median with interquartile range, *n* = 6. *p < 0.05, **p < 0.01, ***p < 0.001, ****p < 0.0001 vs. control

The first series of experiments measured whether fentanyl alone can induce platelet aggregation in whole blood. Based on the data collected for the AUC variable, the mean level of spontaneous platelet aggregation in blood (i.e., when saline was added instead of agonist) was 9.3 ± 2.8 U. Fentanyl induced a slight increase in platelet aggregation (10.7 ± 2.3 U and 10.7 ± 3.7 U, respectively, for 2 and 2000 ng/ml fentanyl; however, the differences were not statistically significant compared to the spontaneous response. On the other hand, stimulation of blood platelets with 2 µM ADP led to four-fold increase of platelet aggregation (*p* = 0.02) over the non-activated platelets (Fig. 1A, RM ANOVA with G–G correction, *F*_1.077, 5.384_ = 18.76, *p* = 0.006).

Since fentanyl itself was shown not to induce platelet aggregation in whole blood, the next experimental runs assessed whether fentanyl is able to potentiate platelet aggregation in response to 2 µM ADP. Fentanyl was shown to have no effect on platelet aggregation stimulated by ADP, either when it was added to blood together with the agonist (Fig. 1B, Friedman’s ANOVA, *χ*^2^ = 12.56, df = 3, *p* = 0.0013) or when fentanyl was preincubated with blood for 15 min before adding the agonist (Fig. 1C, RM ANOVA, *F*_2, 10_ = 1.564, NS). In contrast, increased platelet aggregation was seen after platelet stimulation with ADP with the addition of 10 µM adrenaline: the AUC was increased by 60% (*p* = 0.0036) compared to control (Fig. 1B).

To test whether fentanyl has the ability to reduce or negate the anti-aggregatory effects of oral P2Y_12_ inhibitors, the blood samples were incubated with prasugrel metabolite in the presence of fentanyl and ADP-induced platelet aggregation was measured. The mean platelet aggregation induced by 5 µM ADP was 52.2 ± 6.4 U (*n* = 12). Prasugrel metabolite markedly decreased ADP-induced platelet aggregation by 13% at 1.3 µM (Fig. 1D, RM ANOVA, *F*_3, 15_ = 14.30, *p* = 0.0001) and by 27% at 2 µM (Fig. 1E, RM ANOVA, *F*_3, 15_ = 25.20, *p* < 0.0001), and this response remained unchanged in the presence of fentanyl (Fig. 1D‒E).

### Effect of fentanyl on platelet aggregation in platelet-rich plasma

It was first assessed whether fentanyl alone can induce platelet aggregation in platelet-rich plasma. The amplitude (*A*_max_) and AUC values of platelet aggregation measured in PRP in response to 2 µM ADP were significantly increased from 2.8 ± 0.8% and 12.8 ± 8.6 U in vehicle-treated PRP (control) to 32.2 ± 15.9% (*p* = 0.0318; detected by RM ANOVA with G‒G correction, F_1.028, 4.112_ = 16.03, *p* = 0.015) and 213.9 ± 178.1 U (*p* < 0.01; evaluated by Friedman’s ANOVA, *χ*^2^ = 9.96, df = 3, *p* = 0.0087). No changes were observed after adding fentanyl instead of ADP as compared with control samples (*A*_max_ and AUC were 3.4 ± 2.7% and 15.2 ± 17.2 U for 2 ng/ml fentanyl and 3.2 ± 2.3% and 16.5 ± 18.6 U for 2000 ng/ml fentanyl, *n* = 5).

**Fig. 2:**
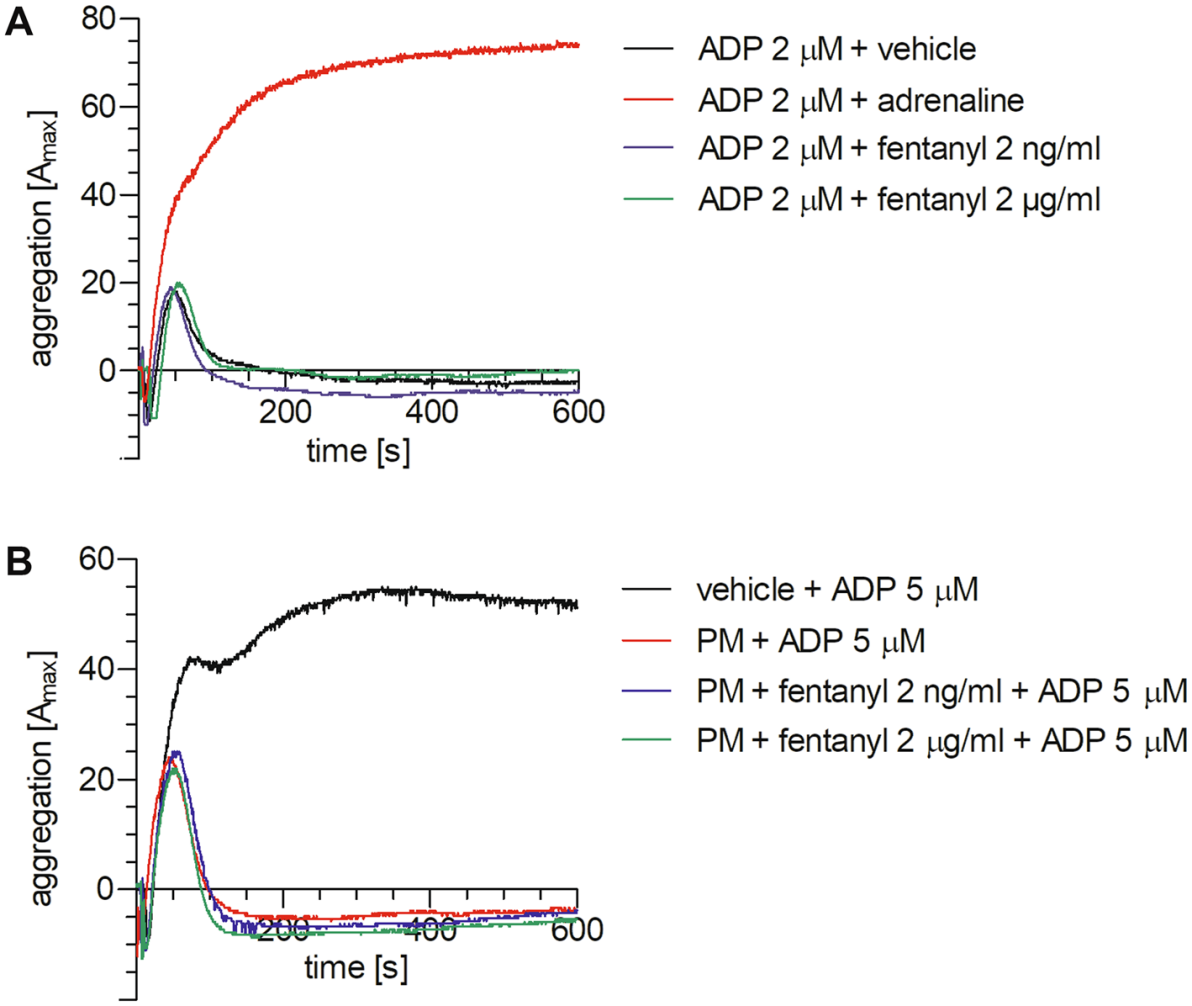
Representative curves of ADP-induced platelet aggregation measured by LTA. The effect of fentanyl on agonist-induced platelet function was measured in PRP, in the absence or presence of 1.3 µM prasugrel metabolite (PM). The example data show platelet response to fentanyl added to PRP with subthreshold concentration of ADP (**A**) or preincubated with PM before stimulation of PRP with 5 µM ADP

The experiments aimed at determining the effects of fentanyl on ADP-induced platelet aggregation in PRP in the absence and presence of prasugrel metabolite were conducted in pairs, where each sample was run with the appropriate control. As shown in Table 1, fentanyl used at 2 or 2000 ng/ml produced little changes in comparison with control (ADP alone), either when it was preincubated with blood for 15 min before adding the agonist (fentanyl 2 ng/ml: for *A*_max_ *t*_4_ = 2.535, NS and for AUC *t*_4_ = 1.37, NS; fentanyl 2000 ng/ml: for *A*_max_ *t*_4_ = 0.3015, NS and for AUC *t*_4_ = 0.7614, NS), or added to PRP together with the agonist (fentanyl 2 ng/ml: for *A*_max_ *t*_4_ = 0.3054, NS and for AUC *t*_4_ = 1.2277, NS; fentanyl 2000 ng/ml: for *A*_max_ *T*_5_ = 4, NS and for AUC *t*_4_ = 0.3788, NS). In contrast, 10 µM adrenaline substantially increased platelet response to 2 µM ADP (*A*_max_: *t*_4_ = 11.37, *p* = 0.0003; AUC: *t*_4_ = 10.01, *p* = 0.0006). Furthermore, prasugrel metabolite (1.3 µM) was found to markedly decrease platelet aggregation induced by 5 µM ADP (*A*_max_: *t*_4_ = 6.315, *p* = 0.0032; AUC: *t*_4_ = 4.037, *p* = 0.0156). However, fentanyl at the concentration of 2 or 2000 ng/ml did not influence the action of prasugrel metabolite in ADP-stimulated platelet-rich plasma (Table 1, fentanyl 2 ng/ml: for *A*_max_ *t*_4_ = 0.5345, NS and for AUC *T*_5_ = 3.00, NS; fentanyl 2000 ng/ml: for *A*_max_ *t*_4_ = 0.3430, NS and for AUC *t*_4_ = 1.442, NS). Representative curves of platelet aggregation are shown in Fig. 2.

**Table 1:** The effect of fentanyl on ADP-induced platelet aggregation in PRP in the absence and presence of prasugrel metabolite

	*A*_max_ [%]		AUC [U]	
	Control sample	Test sample	Control sample	Test sample
Experiments with ADP				
Fentanyl 2 ng/ml (incubation) + ADP 2 µM	24.8 ± 6.3	21.8 ± 8.0	54.9 ± 46.2	67.3 ± 66.4
Fentanyl 2 µg/ml (incubation) + ADP 2 µM	25.9 ± 10.3	25.7 ± 11.5	95.2 ± 131.0	85.6 ± 109.1
Fentanyl 2 ng/ml + ADP 2 µM	20.8 ± 7.5	20.2 ± 3.4	82.7 ± 106.6	50.1 ± 56.4
Fentanyl 2 µg/ml + ADP 2 µM	22.2 ± 3.3	22.8 ± 2.9	77.9 ± 82.6	72.1 ± 81.6
Adrenaline 10 µM + ADP 2 µM	26.4 ± 7.0	84.4 ± 8.9***	139.9 ± 104.3	700.8 ± 80.9***
Experiments with ADP and prasugrel metabolite				
PM 1.3 µM (incubation) + ADP 5 µM	63.0 ± 19.2	34.8 ± 18.8**	512.9 ± 237.7	208.7 ± 224*
PM 1.3 µM + fentanyl 2 ng/ml (incubation) + ADP 5 µM	31.4 ± 13.7	30.8 ± 11.4	100.7 ± 106.7	76.1 ± 76.8
PM 1.3 µM + fentanyl 2 µg/ml (incubation) + ADP 5 µM	31.0 ± 16.3	31.2 ± 15.4	147.1 ± 184.3	110.6 ± 135.4

Data are presented as mean and standard deviation (*n* = 5). Experiments were performed in pairs, where each sample was run with the appropriate control. Differences between the groups were analyzed using Student’s *t* test or Wilcoxon test

^*^p < 0.05, ^**^p < 0.01, ^***^p < 0.001 vs. control sample

Further experiments were carried out in PRP assessing whether high concentration fentanyl (2000 ng/ml) is able to enhance the platelet response to various ADP concentrations and/or neutralize the prostaglandin E_1_-mediated inhibition of platelet aggregation. Platelet aggregation increased from 2 to 91% in response to increasing exogenous ADP concentrations (0.1‒20 µM) (*n* = 5); however, this was not influenced by the addition of fentanyl as no significant differences were observed between the dose–response curves for ADP alone and ADP with fentanyl (Supplementary Fig. S1, evaluated by extra sum-of-squares *F* test: for *A*_max_ *F*_4, 90_ = 0.02455, NS and for AUC *F*_4, 90_ = 0.02114, NS). Furthermore, PGE_1_, a potent cAMP stimulator, strongly inhibited platelet aggregation induced by 5 µM ADP (decrease was by 70%, *n* = 5, *p* = 0.0002), and this inhibition was not significantly affected by fentanyl (Supplementary Fig. S2, RM ANOVA *F*_2, 8_ = 30.45, *p* = 0.0002).

### Effect of fentanyl on platelet activation

Given that fentanyl did not alter platelet aggregation in whole blood or platelet-rich plasma, the next stage examined whether fentanyl may affect platelet activation. In addition, since fentanyl is highly bound by plasma proteins, the contribution of blood components to the platelet response to fentanyl was determined by measuring platelet activation in blood, PRP and in suspensions of isolated platelets.

**Fig. 3:**
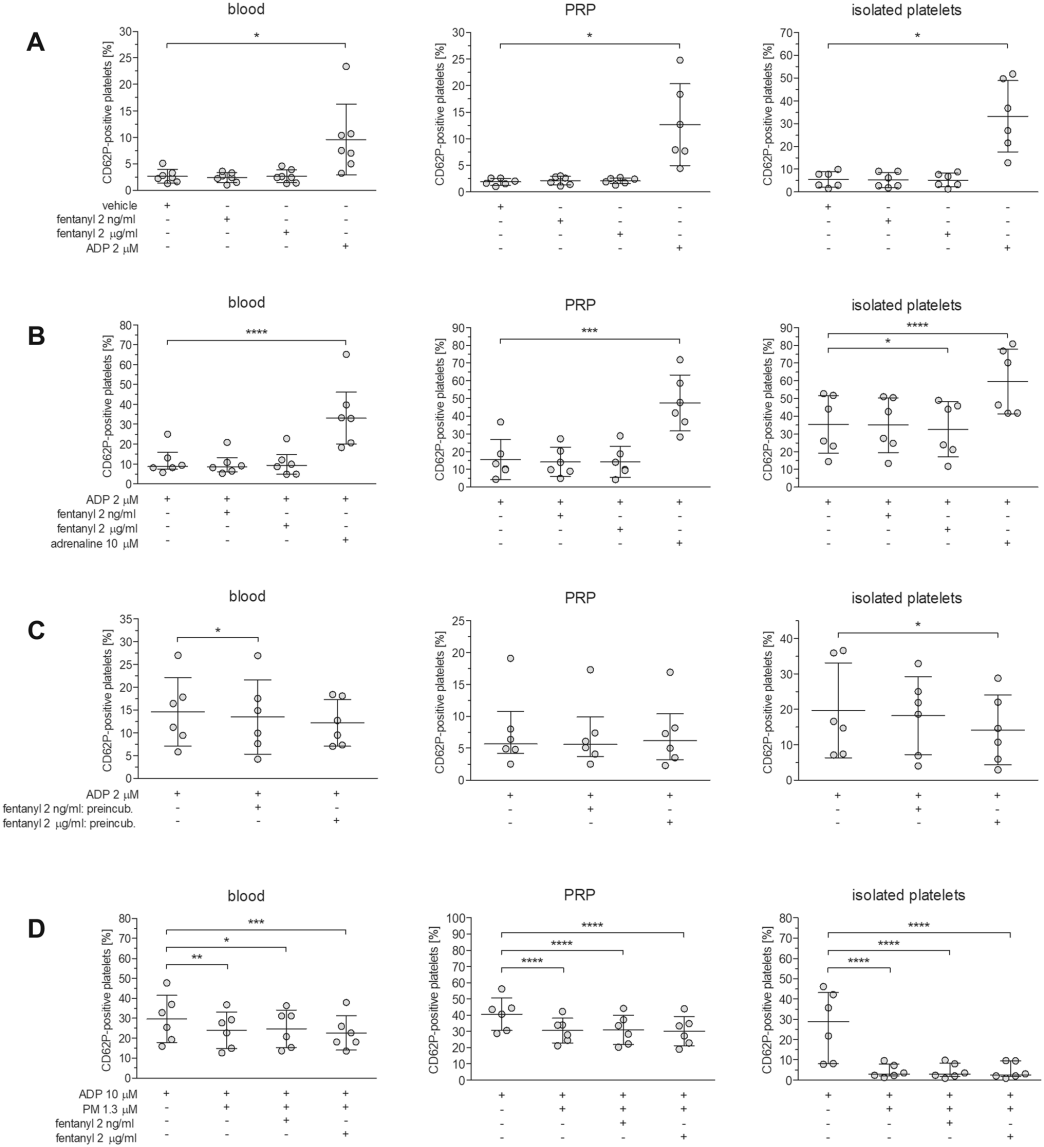
Effect of fentanyl on platelet activation. The influence of 2 or 2000 ng/ml fentanyl on platelet function was measured in blood, PRP or suspensions of isolated platelets in the following models: non-stimulated cells (**A**), cells stimulated with 2 µM ADP in combination with fentanyl (**B**), cells stimulated with 2 µM ADP after 15-min preincubation with fentanyl (**C**), and cells stimulated with 10 µM ADP after 15-min preincubation with prasugrel metabolite and fentanyl (**D**). Data with normal distribution (**A**, **B**: PRP/platelets, **C**/**D**: blood/platelets, **D**: blood/PRP) are expressed as mean ± SD; otherwise data are presented as median with interquartile range, *n* = 6‒7. Before statistical analysis, data showing departure from normal distribution were log transformed for normalization. *p < 0.05, **p < 0.01, ***p < 0.001, ****p < 0.0001 vs. the appropriate controls: vehicle (**A**) or ADP (**B**‒**D**)

It was first evaluated whether fentanyl alone can induce platelet activation. Spontaneous platelet activation following stimulation with 2 µM ADP, monitored as P-selectin (CD62) expression on platelet surface, significantly increased from 2.7 ± 1.3 to 9.6 ± 6.7% in blood, from 1.9 ± 0.6 to 12.7 ± 7.7% in PRP and from 5.5 ± 3.4 to 33.2 ± 15.6% in isolated platelet suspensions (Fig. 3A, RM ANOVA with G‒G correction for blood, PRP and suspensions of isolated platelets was *F*_1.020, 6.119_ = 9.743, *p* = 0.0198, *F*_1.009, 5.046_ = 13.13, *p* = 0.0149 and *F*_1.023, 5.114_ = 20.69, *p* = 0.0057, respectively). No changes in platelet activation were found after adding 2 or 2000 ng/ml fentanyl instead of agonist (Fig. 3A).

Next, the ability of fentanyl to enhance platelet activation in response to 2 µM ADP was determined. Unlike adrenaline, fentanyl did not enhance platelet activation when added to blood, PRP or isolated platelets with ADP (Fig. 3B, blood: RM ANOVA, *F*_3, 15_ = 120.6, *p* < 0.0001; PRP: RM ANOVA with G‒G correction, *F*_1.321, 6.606_ = 96.27, *p* < 0.0001; platelets: RM ANOVA with G‒G correction, *F*_1.288, 6.438_ = 153.4, *p* < 0.0001). Instead, preincubation of blood, PRP or isolated platelets suspensions with fentanyl led to slight decreases in ADP-induced platelet activation, by mean values of 7% at 2 ng/ml fentanyl and by 17% at 2000 ng/ml fentanyl. Significant differences were found after incubation of fentanyl with blood (Fig. 3C, reduction of platelet activation with 2 ng/ml fentanyl by 8% (*p* = 0.0231) compared to control, RM ANOVA with G‒G correction, *F*_1.041, 5.204)_ = 1.931, *p* = 0.2224) or with suspensions of isolated platelets (Fig. 3C, reduction of platelet activation with 2000 ng/ml fentanyl by 28% (*p* = 0.0363) compared to control, RM ANOVA, *F*_2, 10_ = 4.075, *p* = 0.0508).

Finally, it was tested whether fentanyl has ability to antagonize the effects of prasugrel metabolite in blood, PRP, and in suspensions of isolated platelets. As shown in Fig. 3D, 10 µM ADP induced considerable increases in platelet activation (up to 29.7 ± 11.9% in blood, 40.7 ± 10.1% in PRP, and 27.0 ± 16.9% in suspensions of isolated platelets, *n* = 6). Preincubation with 1.3 µM prasugrel metabolite resulted in reduced platelet activation in response to ADP by 19% (*p* = 0.0058) in blood, 25% (*p* < 0.0001) in PRP and by 84% (*p* < 0.0001) in isolated platelets (Fig. 3D, RM ANOVA for blood, PRP and suspensions of isolated platelets was *F*_3, 15_ = 9.219, *p* = 0.0011, *F*_3, 15_ = 74.68, *p* < 0.0001 and *F*_3, 15_ = 88.14, *p* < 0.0001, respectively). However, no differences in ADP-induced platelet activation were noted in blood, PRP or isolated platelets pretreated with PM and fentanyl at 2 or 2000 ng/ml compared with control (blood, PRP or isolated platelets incubated with PM alone) (Fig. 3D).

### Analysis of the interactions between fentanyl or prasugrel metabolite and human carrier proteins

**Fig. 4:**
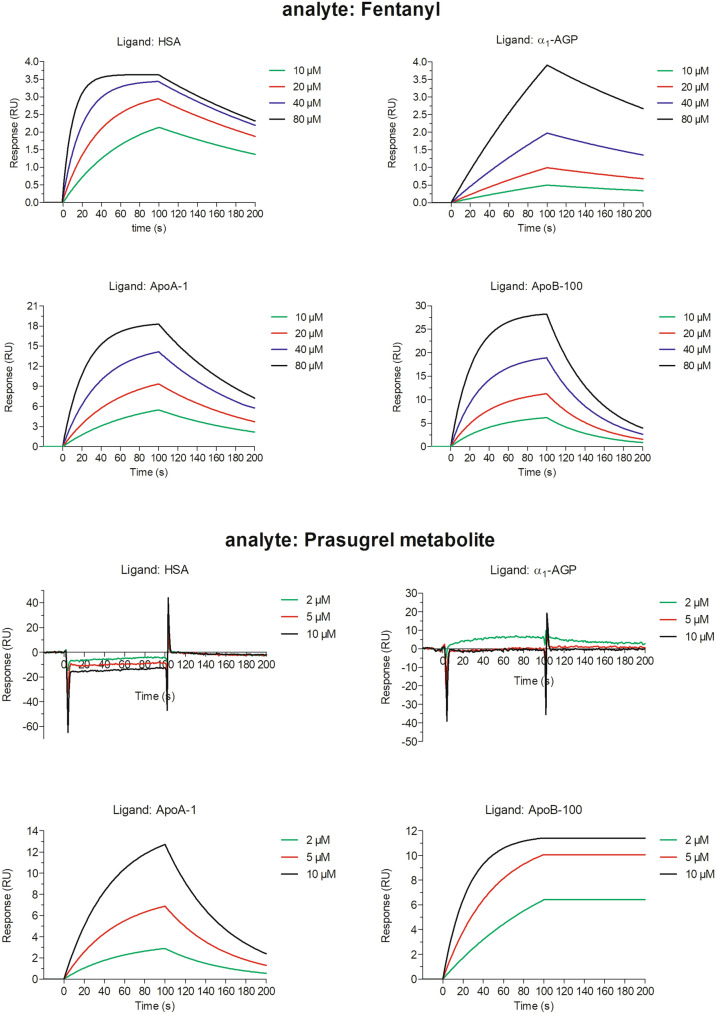
Representative SPR sensograms showing the interactions of fentanyl or prasugrel metabolite with HSA, α_1_-AGP, ApoA-1, or ApoB-100. The proteins were immobilized to a dextran-coated gold surface. Injection of analyte (10, 20, 40 80 μM fentanyl or 2, 5, 10 μM prasugrel metabolite) produced a signal change that was directly proportional to the mass of bound drug molecules, expressed in resonance units (RU). Further details are given in Materials and Methods

The kinetic studies suggest that fentanyl was able to bind to all the examined human carrier proteins (HSA, α_1_-AGP, ApoA-1 and ApoB-100) (Table 2). The strongest and most stable interactions were found between fentanyl and ApoA-1 with K_D_ < 1 × 10^–9^ M, i.e., a value comparable with those observed for the interactions between a receptor and a ligand or between a monoclonal antibody and its specific epitope. In the case of other examined plasma proteins, the interactions with fentanyl were significantly weaker, with dissociation equilibrium constant values varying between 10^–5^ and 10^–4^ M, i.e., the level of the interactions between the cell adhesion molecules, as well as of the major histocompatibility complex molecules binding with T-lymphocytes, or of the phospho-peptides interactions with SH2-domains. In contrast, the complexes of fentanyl with HSA were formed faster than those between fentanyl and the remaining proteins (Fig. 4).

**Table 2:** Summary of kinetic constants and binding affinities for the interactions of fentanyl and prasugrel metabolite with human plasma proteins assayed by SPR

Ligand	*k*_*a*_ (M^−1^ × s^−1^)	*k*_*d*_ (s^−1^)	*K*_*A*_ (M^−1^)	*K*_*D*_ (M)	*χ*^*2*^
	Analyte: fentanyl				
HSA	4.76 ± 6.62 × 10^3^	2.02 ± 1.41 × 10^−2^	2.24 ± 1.82 × 10^5^	1.26 ± 1.62 × 10^−5^	0.47 ± 0.12
α_1_-AGP	5.89 ± 5.47 × 10^2^	4.22 ± 4.44 × 10^−3^	2.69 ± 4.66 × 10^8^	2.16 ± 3.68 × 10^−4^	0.19 ± 0.06
ApoA-1	3.42 ± 0.22 × 10^2^	1.18 ± 1.15 × 10^−6^	6.81 ± 7.08 × 10^8^	3.50 ± 3.56 × 10^−9^	0.86 ± 0.06
ApoB-100	1.34 ± 0.16 × 10^2^	2.52 ± 0.25 × 10^−2^	5.41 ± 1.10 × 10^3^	1.91 ± 0.43 × 10^−4^	0.95 ± 0.34

	Analyte: prasugrel metabolite				
HSA	No binding				
α_1_-AGP	No binding				
ApoA-1	2.09 ± 1.43 × 10^3^	5.57 ± 9.64 × 10^−3^	1.17 ± 2.00 × 10^10^	1.20 ± 2.08 × 10^−5^	0.43 ± 0.18
ApoB-100	2.57 ± 0.98 × 10^3^	4.96 ± 0.84 × 10^−6^	5.48 ± 2.99 × 10^8^	2.17 ± 0.96 × 10^−9^	0.84 ± 0.29

Data are presented as mean ± SD, *n* = 3

Prasugrel metabolite showed no binding affinity to HSA or α_1_-AGP, but it interacted with both apolipoproteins. The complexes of PM with ApoB-100 were formed faster than with ApoA-1, as can be concluded both from the shape of the sensorgram curve in the association phase (Fig. 4) and from the calculated K_A_ values (Table 2). Furthermore, the shape of dissociation curve and the obtained K_D_ suggest that the complexes of PM with ApoB-100 appeared to be stronger and more stable than those of PM and ApoA-1 (Table 2, Fig. 4).

The goodness of fit value, as indicated by the mean Chi-square value (*χ*^2^), was below 1 for all the SPR experiments (Table 2), which suggests that the obtained kinetic parameters are highly reliable.

### Molecular docking

To provide a more detailed information on the interaction of fentanyl with HSA, α_1_-AGP, and ApoA-1, *in silico* computational experiments were performed with the use of molecular docking. The general results of all docking simulations, namely the values of the Gibbs free energy of binding, dissociation and association contains, as well as the number of identified hydrogen bonds, π-cation interactions, and π–π interactions, are summarized in Table 3; this shows the values obtained for the most energetically favorable conformations, chosen out of 60 performed conformers for each docking simulation.

**Table 3:** Molecular docking analysis of the interactions between fentanyl and plasma proteins

Protein/PDB ID and grid box setting	Gibbs free energy of binding (*ΔG*_*b*_; kcal/mol)	Dissociation constant (*K*_*D*_; nM)	Association constant (*K*_*A*_; nM^−1^)	Number of:		
				Hydrogen bonds	π–cation interactions	π–π interactions
HSA/2BXD: Sudlow site I	− 8.28 × 10^0^	8.49 × 10^2^	1.18 × 10^−3^	2	0	0
HSA/2BXG: Sudlow site II	− 8.19 × 10^0^	9.97 × 10^2^	1.00 × 10^−3^	1	0	0
HSA/2VUE: drug site III	− 9.21 × 10^0^	1.79 × 10^2^	5.60 × 10^−3^	0	0	0
α_1_-AGP/3APU: blind	− 6.86 × 10^0^	9.31 × 10^3^	1.07 × 10^−4^	0	0	0
α_1_-AGP/3APU: site specific	− 8.43 × 10^0^	6.67 × 10^2^	1.50 × 10^−3^	1	0	0
α_1_-AGP/3KQ0: blind	− 6.41 × 10^0^	2.02 × 10^4^	4.96 × 10^−5^	0	0	0
α_1_-AGP/3KQ0: site specific	− 8.63 × 10^0^	4.74 × 10^2^	2.11 × 10^−3^	0	0	1
α_1_-AGP/7OUB: blind	− 6.97 × 10^0^	7.72 × 10^3^	1.30 × 10^−4^	0	0	0
α_1_-AGP/7OUB: site specific	− 8.19 × 10^0^	9.95 × 10^2^	1.00 × 10^−3^	0	0	0
ApoA-1/1AV1: blind	− 4.88 × 10^0^	2.64 × 10^5^	3.79 × 10^−6^	0	0	0
ApoA-1/1AV1: site specific	− 6.00 × 10^0^	4.01 × 10^4^	2.49 × 10^−5^	0	0	0
ApoA-1/2N5E: blind	− 4.58 × 10^0^	4.37 × 10^5^	2.28 × 10^−6^	0	0	0
ApoA-1/2N5E: site specific	− 6.12 × 10^0^	3.27 × 10^4^	3.06 × 10^−5^	0	0	0
ApoA-1/3R2P: blind	− 5.80 × 10^0^	5.60 × 10^4^	1.79 × 10^−5^	0	0	1
ApoA-1/3R2P: site specific	− 7.54 × 10^0^	2.99 × 10^3^	3.34 × 10^−4^	0	0	1

To characterize the interactions of fentanyl with HSA, the docking simulations were performed to verify whether fentanyl can bind the common drug binding sites within the HSA molecule: Sudlow site I, Sudlow site II, and drug site III. The Gibbs free energy of binding values suggested that the interaction of fentanyl with all three examined drug binding sites is energetically favorable, with the most negative value in the case of drug site III, although this was slightly lower than the others (Table 3).

For site-specific docking simulations of α_1_-AGP1 (3APU, 3KQ0, 7OUB) and ApoA-1 (1AV1, 2N5E, 3R2P), the grid boxes were defined to include the residues found to be in close contact with fentanyl based on blind docking. All the residues in close contact with fentanyl are shown in Table 4. In the case of α_1_-AGP1 interactions with fentanyl, the involved residues represent one primary ligand binding site of the glycoprotein molecule with high affinity and low capacity, namely lobe I. The results of the molecular docking experiments performed with ApoA-1 differed depending on the crystal structure used for the study; however, fentanyl preferentially interacted with helical regions of the ApoA-1 molecule.

**Table 4:** Amino acid residues in plasma proteins identified by molecular docking to be in close contact with the fentanyl molecule

Protein/PDB#	Grid box setting	Residues in close contact
HSA/2BXD	Sudlow site I	LYS199 PHE211 TRP214 ALA215 LEU219 ARG222 LEU238 ARG257 LEU260 ALA261 ILE264 SER287 ILE290 ALA291
HSA/2BXG	Sudlow site II	LEU387 ILE388 ASN391 CYS392 PHE395 PHE403 ARG410 TYR411 GLY434 CYS438 LEU453 LEU457 PHE488 SER489
HSA/2VUE	Drug site III	LEU115 ARG117 PRO118 MET123 TYR138 ILE142 PHE157 TYR161 LEU185 GLY189
α_1_-AGP/3APU	Blind	PHE32 TYR37 SER40 VAL41 VAL88 ARG90 GLU92 PHE112 TYR127
α_1_-AGP/3KQ0	Blind	PHE32 TYR37 VAL41 ARG90 HIS97 ALA99 LEU112 PHE114
α_1_-AGP/7OUB	Blind	GLU64 GLN66 VAL88 ARG90 GLU92 HIS97 PHE112 SER114
α_1_-AGP/3APU	Site specific	TYR27 ILE44 THR47 PHE49 GLU64 GLN66 VAL88 SER89 ARG90 HIS97 ALA99 PHE112 SER114 SER125 TYR127
α_1_-AGP/3KQ0	Site specific	PHE32 TYR37 LEU79 ILE88 SER89 ARG90 VAL92 HIS97 LEU112 PHE114 ASN117 ASN121 TYR127
α_1_-AGP/7OUB	Site specific	TYR37 GLU64 GLN66 ASN75 VAL88 SER89 ARG90 GLU92 HIS97 VAL98 ALA99
ApoA-1/1AV1	Blind	GLU70 PHE71 ASN74 LEU75 GLU78 THR79 LEU82
ApoA-1/2N5E	Blind	VAL67 THR68 PHE71 GLU235 LYS239 LEU240
ApoA-1/3R2P	Blind	VAL30 TYR100 LEU101 PHE104 LYS107
ApoA-1/1AV1	Site specific	PHE71 ASN74 LEU75 GLU78 THR79 LEU82
ApoA-1/2N5E	Site specific	GLU62 GLN63 GLY65 VAL67 THR68 GLU235 TYR236 LYS239 LEU240
ApoA-1/3R2P	Site specific	GLY26 TYR29 VAL30 TRP50 VAL97 TYR100 LEU101 PHE104 LYS107 TRP108

The simulation performed with the 2BXD structure of HSA at Sudlow site I revealed two hydrogen bonds formed between two hydrogens of ARG222 guanidinium group serving as H-bond donors (HH12 in the case of the first H-bond, and HH22 in the case of the second) and an oxygen atom from the fentanyl molecule (acceptor). When docking was performed with the 2BXG structure of HSA at Sudlow site II, one hydrogen bond was identified between ARG410 (HH22 hydrogen atom of ARG410 guanidinium group, donor) and the oxygen atom from the fentanyl molecule (acceptor). The site-specific docking simulation of the 3APU structure of α_1_-AGP1 identified one hydrogen bond between SER125 (HG hydrogen atom from the hydroxyl group of hydroxymethyl side chain of SER125, donor) and the oxygen atom from the carbonyl group (position: C16-O17) of fentanyl (acceptor).

Several π–π interactions were detected in the case of three simulations. During the site-specific docking with 3KQ0 structure of α_1_-AGP1, a π–π interaction was formed between N-phenyl ring of fentanyl and benzyl ring from PHE114. Both blind and site-specific docking of ApoA-1 structure with 3R2P PDB ID resulted in the π–π interaction detected between the benzyl ring of fentanyl and benzyl ring of PHE104.

**Fig. 5:**
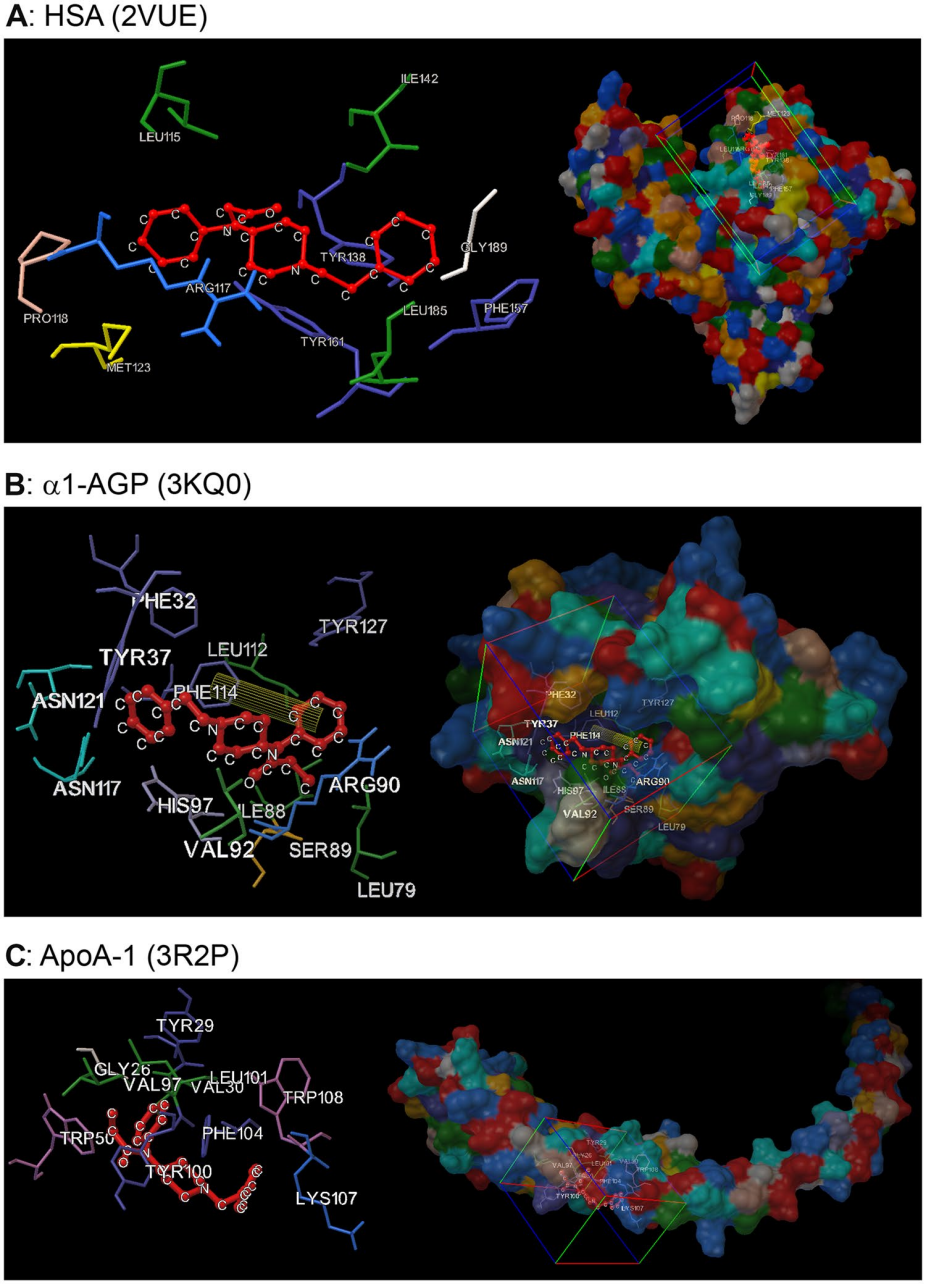
Docking of fentanyl to the selected plasma carrier proteins. The analysis was performed for the proteins with the available X-ray structure: HSA, α_1_-AGP, and ApoA-1. The most energetically favorable conformations for each protein are shown. One most favorable PDB structure of each protein was chosen. **A** Fentanyl was docked to bilirubin binding site, so called “drug site III” within the 2VUE crystal structure of HSA. **B** and **C** Fentanyl was docked to a binding site of α_1_-AGP (3KQ0) or ApoA-1 (3R2P) protein crystal structure that was previously identified by blind docking. The binding sites at two different magnifications are shown. Cuboids (on the right) indicate the location and size of the grid box. The yellow cylinder joining the benzene rings of fentanyl and of LEU112 residue

No hydrogen bonds and no π–π interactions were detected in the case of the following analyses: docking of HSA/2VUE at drug site III, blind docking of α_1_-AGP1/3APU, blind docking of α_1_-AGP1/3KQ0, blind and site-specific docking of α_1_-AGP1/7OUB, blind and site-specific docking of ApoA-1/1AV1, and ApoA-1/2N5E. In the case of the studied interactions, the binding process was mainly driven by van der Waals forces, hydrogen bonds, and desolvation (observations based on raw data of docking analyses, not shown). The results of all docking simulations did not reveal any π-cation interactions. Figure 5 illustrates the most energetically favorable interactions, i.e., with the most negative values of Gibbs free energy of binding, of fentanyl with each protein: 2VUE for HSA, 3KQ0 for α_1_-AGP1, and 3R2P for ApoA-1. More detailed results of molecular docking simulations are presented in Supplementary Materials.

## Discussion

Limited data exists on the interactions of fentanyl with blood components, including blood platelets and plasma proteins. Also, the causes of impaired responsiveness to antiplatelet drugs by fentanyl remain unclear. This in vitro study aimed to evaluate the ability of fentanyl to: i) stimulate platelet function, ii) enhance agonist-induced platelet function, iii) decrease platelet sensitivity to antiplatelet drug in agonist-stimulated platelets, and iv) interact with selected carrier proteins. The results presented here indicate that fentanyl itself applied under different in vitro experimental conditions at therapeutic and supratherapeutic concentrations has no potential to stimulate platelet function or potentiate platelet response to stimulatory agents, such as ADP. Unprecedentedly, our data indicate that fentanyl directly does not affect platelet susceptibility to inhibitory agents, such as prasugrel metabolite. As regards the interactions of fentanyl with plasma proteins, our data demonstrate direct interactions of fentanyl with α_1_-AGP, ApoA-1, and ApoB-100, highlighting their role in the transport of fentanyl in blood.

### Study of interactions of fentanyl with blood platelets

The effect of fentanyl on platelet function was first evaluated in whole blood using the Multiplate system, which is one of the most widely used platelet function analyzers [35]. Recently, this tool has been recommended for monitoring platelet function in patients on antiplatelet therapy undergoing CABG surgery [36]. The action of the opioid on platelet aggregation in blood of healthy subjects was evaluated in non-activated platelets and platelets stimulated with ADP that was used at subthreshold and high concentrations, depending on the experimental model. We hypothesized that fentanyl may elicit stimulatory effects in human platelets, so in most experiments low ADP concentration was used; however, higher ADP concentration was used in experiments with prasugrel metabolite (PM) in order to observe the effects of both P2Y_12_ inhibitor and fentanyl. As expected, platelet aggregation depended on agonist concentration, as the mean platelet aggregation induced by 2 μM ADP was over 1.3-fold lower than that with 5 μM ADP. These findings are in line with the results by Peerschke et.al., who established the reference ranges for platelet aggregation in response to various agonists using blood of healthy donors collected into different anticoagulants [37]; the mean platelet aggregation for citrate-anticoagulated blood in response to 6.5 μM ADP was estimated to be 68.6 U, indicating that platelet aggregation in response to 6.5 μM ADP can be 1.3-fold higher than that observed in response to 5 μM ADP. Fentanyl alone or added to blood in combination with ADP, at therapeutic and supratherapeutic concentrations, did not exert any effect on platelet aggregation; most importantly, it had also no influence on the inhibition of platelet aggregation by prasugrel metabolite in ADP-stimulated blood samples. Few reports have explored the direct interactions between fentanyl and platelets to date. None of them, however, have explored the effect of fentanyl on platelet sensitivity to antiplatelet drugs under in vitro conditions. In a study of the effects of fentanyl on platelet function in whole blood, Palorali et al. [38] also found that fentanyl used at therapeutic concentration (1 ng/ml) has no effect on spontaneous or agonist-induced platelet activation measured in vitro in the blood of healthy volunteers and ex vivo in patients undergoing angioplasty [38].

Opioids may regulate the functions of immune cells, including proliferation, chemotaxis, trafficking, cytokine synthesis and secretion [39]. Considering the potential interactions of fentanyl with leukocytes in blood, we examined the action of fentanyl on platelet function in the absence of other blood cells, i.e., optical aggregometry (LTA) of platelet-rich plasma, which is regarded as the gold standard for assessing the platelet response to agonists. Similarly to results obtained in blood, we found no differences in platelet aggregation in the presence of fentanyl compared to the controls, as regards basal response (with no agonist) or platelet response to ADP, with or without prasugrel metabolite. In addition, fentanyl was found not to have an antagonistic effect on the anti-aggregatory action of PGE_1_—a potent cAMP stimulator. The influence of fentanyl and other anesthetic agents on the agonist-induced platelet aggregation in PRP has been studied previously. In line with our results, fentanyl was not found to influence ADP- or collagen-stimulated platelet aggregation in patients undergoing major operations and anaesthetized with fentanyl, nor were any effects observed on thromboelastography parameters [40]. These findings were confirmed in another in vitro study performed in PRP of healthy volunteers, showing that platelet aggregation evoked by ADP or collagen remains unchanged by the addition of fentanyl at concentrations up to 50 ng/ml [41].

The effectiveness of fentanyl in modulating blood cell function may be influenced by its high plasma protein binding potential [12]. Therefore, we implemented flow cytometry to assess the influence of fentanyl on platelet activation in suspensions of isolated platelets, along with in PRP and whole blood. Flow cytometry is a useful tool in monitoring cell function and allowing detection of small changes in platelet function [20, 42]. Unfortunately, we did not observe any increased platelet activation following platelet incubation with fentanyl: the opioid did not exert stimulatory activity on spontaneous- and ADP-induced platelet activation, regardless of the type of biological sample. Instead, fentanyl was shown to markedly inhibit ADP-induced platelet activation. These findings, however, are inconclusive and seem to be clinically insignificant as the inhibitory effect of fentanyl was mainly observed in samples of isolated platelets containing fentanyl at supratherapeutic concentration. Also, at therapeutic and supratherapeutic concentrations, fentanyl failed to abolish the inhibitory effect of prasugrel metabolite following ADP-induced platelet activation. Taken together, our data are consistent with those of Gruba et al. [5], who report that stimulation of platelet opioid receptors in wildtype mice by DAMGO, DPDPE, or U-50488, which target the μ-, δ-, and κ-opioid receptors, does not lead to activation of isolated platelets. Likewise, the δ-granule secretion from thrombin-stimulated platelets has been shown to be unaffected by opioid receptor agonists. Based on all collected data, it has been postulated that opioids, such as morphine or ketamine, do not significantly affect platelet function [5].

Overall, although platelet response has been shown to be affected by some anesthetics, the results presented here indicate that it is not the case for fentanyl, which has no potential to stimulate (or co-stimulate) platelet function, either when acting alone or in conjunction with platelet agonist. Also, fentanyl was shown not to be effective in modulating the effects of prasugrel metabolite in ADP-stimulated platelets. Such observations may suggest that the blunted responsiveness to oral P2Y_12_ inhibitors, such as prasugrel, observed in patients undergoing percutaneous coronary intervention does not appear to result from the direct interactions between fentanyl and blood platelets.

### Interactions of fentanyl with plasma proteins

The ability of fentanyl to regulate platelet function may strongly depend on the presence of plasma proteins, which can bind up to 80% of circulating fentanyl [12]. The interactions of fentanyl with plasma proteins, particularly with HSA and α_1_-AGP, are widely documented, but the results are not clear and sometimes contradictory, mostly in terms of specificity. Other blood components, such as erythrocytes, lipoproteins, and α-, β-, and γ-globulins, are also potential binding partners for fentanyl. Hence, this opioid drug may be transported in the blood by more than one plasma protein. Our study therefore examines the binding of fentanyl to some common plasma proteins, including HSA, α_1_-AGP, ApoA-1, and ApoB-100, using two approaches: laboratory in vitro experiments performed by surface plasmon resonance (SPR) and in silico simulation by molecular docking.

Our SPR results show that fentanyl was able to bind to all the examined proteins, with the strongest and most stable interactions being found between fentanyl and ApoA-1. The dissociation constant (K_D_) of the fentanyl‒ApoA-1 complex was significantly marked against the background of those with other examined plasma proteins, which were several orders of magnitude weaker. The sensorgram shapes suggest, however, that complexes of fentanyl with HSA were formed faster than those between fentanyl and the remaining plasma proteins. Contrary to fentanyl, prasugrel metabolite showed a selective mode of binding: it bound to apolipoproteins, but not to HSA or α_1_-AGP. The lack of binding of PM to HSA was the most surprising finding, since previous studies have reported extensive binding of prasugrel metabolite (R-138727) to HSA with a binding ratio of 98% [43]. This difference could be due to the approach used to assess the binding. Previously, PM binding to albumin has been confirmed by ultracentrifugation, in which a carrier protein is in solution. The present study used a SPR technique, where a carrier protein is immobilized on the sensor surface to assess the interactions between molecules. Assuming that only a weak interaction occurs between prasugrel metabolite and albumin, then it is possible that it may not be detected with SPR as this technique is recommended for moderate and high-affinity systems [44].

The fentanyl molecule is basic with a pKa value of 8.43, highly lipophilic/hydrophobic and while present in blood, at pH 7.4, about 70–80% of this opioid is bound to plasma proteins with 20–30% remaining free [13, 45, 46]. Hydrophobic interactions between the unionized form of the opioid drugs and the plasma proteins play a crucial role in the degree of binding. The ionized form is bound to a negligible extent, possibly in part by ionic mechanism [13].

The reports on the interactions between fentanyl and α_1_-acid glycoprotein (α_1_-AGP), a key carrier protein for basic and neutral drugs, are ambiguous. Experiments in which α_1_-AGP-bound drugs were displaced by with tributoxyethyl phosphate (TBEP), found the largest contribution to binding to be due to serum albumin; in addition, indirect observations suggested that fentanyl binding to α_1_-AGP did not occur [14]. This study found that fentanyl shares the binding site with aspirin and phenylbutazone in albumin, and with quinidine in lipoproteins. Other papers have, however, suggested that fentanyl interacts with α_1_-AGP, albeit to a lesser extent than other basic drugs [12, 47, 48]. Despite being a basic drug, fentanyl does not appear to bind predominantly to α_1_-AGP, even in the absence of HSA; the fact that the polar metabolite norfentanyl does not appear to bind to α_1_-AGP suggests that such binding most likely occurs at the lipophilic site of HSA [12].

Fentanyl itself has been found to noncompetitively interfere with the binding of another anaesthetic, thiopental, to albumin: the two drugs display different physical and chemical properties [49]. In patients with hyperlipoproteinemia, fentanyl demonstrated enhanced binding to whole plasma; this was attributed to the presence of elevated lipoprotein fractions, since the level of fentanyl binding to albumin was not altered [50]. Fentanyl and its analogs were found to interact with α_1_-AGP in maternal and neonatal plasma [51], with α_1_-AGP and albumin in term and preterm infants [52] and, to a lesser extent, with α- and β-globulin fractions [53]. Fentanyl is predominantly captured in plasma by albumin, with an average of 20 fentanyl molecules bound to a single albumin molecule [54].

Our docking simulations indicate that fentanyl interaction is energetically favorable for all three common drug binding sites within the HSA molecule. However, the interaction with drug site III within the IB subdomain seems to be the most advantageous. This observation partially corresponds with another study, where the top-ranked site for fentanyl was found at the IA–IB domain, containing also the fatty acid and halothane binding sites (FA1/HAL8) [55]. Drug site III, also known as bilirubin binding site, is found in a form of L-shaped pocket located at the entrance of FA1 cavity in subdomain IB [56]. Taking into account its lipophilic characteristics, the fentanyl molecule can preferentially interact with the hydrophobic cleft generated by the packing of helices h8–h10 into a three-helix bundle, which is present within the apolar side chain that closely contacts the drug site III [34]**.** Drug site III was previously shown to bind another hydrophobic drug, lapatinib [57].

HSA molecule contains the single tryptophan residue at position 214 (TRP214), which can be used to measure the inhibition of TRP214 fluorescence, to determine and identify the affinity of compounds that bind near the site [58]. In a study by Zhou et al., fentanyl did not change the intrinsic TRP214 fluorescence signal, suggesting that this is not the binding site for fentanyl [55]. Our docking analysis identified TRP214 as being in close contact only in the case of 2BXD structure, when the grid box was set to cover the Sudlow site I, including TRP214. However, the most energetically favorable interaction was revealed in the case of drug site III, which is not in the proximity of the TRP214 residue.

As such, our results are in a good agreement with those of Zhou and co-workers. They performed the direct binding studies (elution chromatography, tryptophan intrinsic fluorescence measurements, isothermal titration calorimetry) and docking simulations demonstrating that opioids, including fentanyl, share sites with general anesthetics: propofol and halothane. The interaction between opioids and HSA was relatively weaker than that between propofol and HSA. It was also weak in comparison with the reported affinity with the native opioid receptors. As revealed by affinity chromatography, fentanyl displayed a longer retention time as compared with naloxone and morphine, indicating a slightly stronger affinity for HSA. Isothermal titration calorimetry experiments have shown that fentanyl, morphine, and naloxone share at least some common binding sites with both tested anesthetics: propofol and halothane. The heat release for propofol–HSA interactions was almost completely inhibited by fentanyl, indicating that fentanyl may share the same two binding sites as propofol for HSA, i.e., in domain IIIA and IIIB. Docking simulations identified an additional possible fentanyl binding site in domain IIIA as well as the binding site of fatty acids (FA), thyroxine 4, diazepam, halothane (HAL3), propofol (PR1), and ibuprofen. Although the structure of fentanyl shows significant structural differences with that of halothane and propofol, fentanyl shares a site located in domain III of HSA with these two anesthetics [55].

Furthermore, our docking analysis showed that fentanyl preferentially interacted with lobe I, the non-polar ligand binding site of α_1_-AGP, characterized with high affinity for hydrophobic molecules [59]. Lobe I comprises eight stranded β-barrels: from BB1 to BB8 [59]. Most of the residues identified to be in close contact with fentanyl are found in BB5 of the α_1_-AGP1 polypeptide chain (Table 4). A similar mode of hydrophobic interactions was shown for the α_1_-AGP interaction with a hydroxylated form of staurosporine, UCN-01 [60]. Arginine at position 90 seems to be the key residue stabilizing the hydrophobic interactions of α_1_-AGP with ligands (both for fentanyl and UCN-01) and it was identified as the residue in close contact for all our docking simulations.

The docking simulations performed with ApoA-1 differed for each crystal structure used. The residues of 1AV1 structure involved in the interaction with fentanyl belong to the α-helix B that is a part of an N-terminal anti-parallel four-helix bundle domain [61]. In the case of 2N5E structure, the residues in close contact are found in α-helix B of the N-terminal domain, and α-helix F of the two-helix C-terminal domain. Finally, the docking study performed with 3R2P structure revealed that the residues from α-helix A, α-helix B, and α-helix C of the N-terminal domain are involved in the interaction with fentanyl (Table 4). The region of 1AV1 structure that interacted with fentanyl is crucial for the interactions with lipids [62, 63]. In the case of the 2N5E structure, fentanyl bound the region on the edges of helices 1 and 10, i.e., in the region between amino- and carboxy-terminal part of the ApoA-1 molecule, which forms a discoid shape [64]. The 3R2P structure forms a half-cycle, where the N-terminal residues found to interact with fentanyl form a hydrophobic environment that buries the hydrophobic amino acids [65]. These are the first mechanistic or molecular docking studies to be performed on the direct interactions of apolipoproteins with fentanyl.

The lipophilic and hydrophobic properties of fentanyl molecule play key roles in its interactions with plasma proteins. Consequently, fentanyl preferentially binds the apolar, hydrophobic regions of their polypeptide chains. Unexpectedly, this opioid drug formed the strongest and most stable complexes with ApoA-1, whereas the interactions with HSA are most widely documented in the available literature. In contrast, the reports describing the interactions between fentanyl and apolipoproteins are severely limited.

In conclusion, our findings directly demonstrate that fentanyl interacts with albumin, α_1_-AGP, ApoA-1, and ApoB-100 and confirm previous studies showing that fentanyl has a high capacity to bind to plasma proteins. Fentanyl was found to exhibit high specificity for apolipoprotein A-1, thus indicating that plasma high-density lipoproteins may play an important role in the distribution of this drug in the body.

## Supplementary Information

Below is the link to the electronic supplementary material.

Supplementary file1 (PDF 178 KB)

Supplementary file2 (PDF 209 KB)

## Data Availability

All data generated or analyzed during this study are included in this published article and its supplementary information files.

## Abbreviations

ADP: Adenosine 5′-diphosphate
α_1_-AGP: α_1_-Acid glycoprotein
*A*_max_: Maximal aggregation
ApoA-1: Apolipoprotein A-1
ApoB-100: Apolipoprotein B-100
AUC: Area under the curve
cAMP: Cyclic adenosine monophosphate
G‒G correction: The Geisser–Greenhouse correction
HSA: Human serum albumin
LTA: Light transmission aggregometry
PBS: Phosphate-buffered saline
PDB: The Protein Data Bank
PGE_1_: Prostaglandin E_1_
PM: Prasugrel metabolite
PPP: Platelet-poor plasma
PRP: Platelet-rich plasma
RM ANOVA: Repeated measures analysis of variance
SPR: Surface plasmon resonance
