# Supplementary material for: Interactions of fentanyl with blood platelets and plasma proteins: platelet sensitivity to prasugrel metabolite is not affected by fentanyl under in vitro conditions
Source: Pharmacol Rep. 2023 Jan 17;75(2):423–41. doi: 10.1007/s43440-023-00447-7 (PMC10060303; doi:10.1007/s43440-023-00447-7)
Supplement: Supplementary file 1 — Supplementary file1 (PDF 178 KB) [file 43440_2023_447_MOESM1_ESM.pdf]

Interactions of fentanyl with blood platelets and plasma proteins: platelet sensitivity to prasugrel metabolite is not affected by fentanyl under in vitro conditions

SUPPLEMENTARY DATA: interactions of fentanyl with platelets

Abbreviations: c – control sample; F – fentanyl; PM – prasugrel metabolite

Table 1. Raw data obtained from LTA aggregometry: Amax [%]

1 channel	2 channel	1 channel	2 channel	1 channel	2 channel
**the effect of fentanyl alone**					
c	F 2 ng/ml	F 2 µg/ml	ADP 2 µM		
2	1	3	23		
3	2	2	18		
3	3	1	59		
2	3	3	31		
4	8	7	30		
**the effect of fentanyl (preincub.) + ADP 2 µM**					
c	F 2 ng/ml	c	F 2 µg/ml		
21	19	25	25		
31	27	21	21		
16	9	13	11		
30	28	40.5	42.5		
26	26	30	29		
**the effect of fentanyl + ADP 2 µM**					
c	F 2 ng/ml	c	F 2 µg/ml	c	adrenaline 10 uM
16	17	22	24	20	75
20	19	18	20	22	99
17	20	21	23	23	82
34	26	23	20	37	85
17	19	27	27	30	81
**the effect of fentanyl + ADP 5 µM + PM 1.3 µM**					
c	PM	PM	PM + F 2 ng/ml	PM	PM + F 2 µg/ml
55	24	24	25	20	22
80	62	43	40	26	27
33	13	12	15	13	13
72	43	33	31	45	44
75	32	45	43	51	50

Table 2. Raw data obtained from LTA aggregometry: AUC [U]

1 channel	2 channel	1 channel	2 channel	1 channel	2 channel
**the effect of fentanyl alone**					
c	F 2 ng/ml	F 2 µg/ml	ADP 2 µM		
0.6	0.7	9.2	116.7		
17.5	5.9	7	33.4		
10.8	10.9	3	505.1		
11.6	13.9	14.2	210.1		
23.7	44.6	48.9	204.2		
**the effect of fentanyl (preincub.) + ADP 2 µM**					
c	F 2 ng/ml	c	F 2 µg/ml		
17.8	13.1	20.4	48.8		
45	50.2	16.4	14.7		
7.2	2.6	4.6	4.3		
89.1	113.7	314.4	271.6		
115.2	157	120	88.4		
**the effect of fentanyl + ADP 2 µM**					
c	F 2 ng/ml	c	F 2 µg/ml	c	adrenaline 10 uM
9.4	10.3	60.5	90.2	24.3	617.8
46.6	11.7	14.2	13.4	113.7	836.3
16.6	11.2	12.7	23.5	64.7	684.4
268	134	88.1	25.8	258	685.8
72.7	83.5	214.2	207.6	239	679.7
**the effect of fentanyl + ADP 5 µM + PM 1.3 µM**					
c	PM	PM	PM + F 2 ng/ml	PM	PM + F 2 µg/ml
463.9	18.6	18.4	18.4	11.7	14.9
702.8	527.4	211.4	96	21.3	18.8
119	4.2	4	6	4.8	5.3
626.1	346.2	48.7	62.6	354.8	227.3
652.7	147	221.1	197.6	342.7	286.9

Table 3. Data obtained from whole blood aggregometry

1 channel	2 channel	3 channel	4 channel
**the effect of fentanyl alone**			
c	F 2 ng/ml	F 2 µg/ml	ADP 2 µM
8	13	8	44
14	12	14	21
6	8	6	39
11	10	10	21
9	8	10	51
8	13	16	57
**the effect of fentanyl + ADP 2 µM**			

c	F 2 ng/ml	F 2 µg/ml	adrenaline 10 µM
26	25	22	52
46	50	47	59
24	23	24	30
49	57	51	69
21	24	23	45
21	24	23	45

**the effect of fentanyl (preincub.) + ADP 2 µM**		
c	F 2 ng/ml	F 2 µg/ml
24	25	23
45	37	46
22	18	16
56	55	42
22	23	25
68	57	60

**the effect of fentanyl + ADP 5 µM + PM 1.3 µM**			
c	PM	PM + F 2 ng/ml	PM + F 2 µg/ml
59	55	55	48
50	41	42	47
46	41	38	40
50	46	41	40
56	50	47	47
55	42	38	42

**the effect of fentanyl + ADP 5 µM + PM 2 µM**			
c	PM	PM + F 2 ng/ml	PM + F 2 µg/ml
52	33	36	33
46	42	41	36
39	29	29	35
58	43	42	44
61	40	41	43
54	39	38	41

Table 4. Data obtained from flow cytometry

**the effect of fentanyl alone: blood**			
c	F 2 ng/ml	F 2 µg/ml	ADP 2 µM
2.2	2.7	2.6	10.7
2.4	2.4	2.4	10.4
2.8	2.2	2.4	7.5
1.3	1.0	1.4	3.2
5.1	3.3	3.9	23.4
3.3	3.6	4.6	7.0
1.6	1.5	1.3	5.0

the effect of fentanyl alone: PRP			
c	F 2 ng/ml	F 2 µg/ml	ADP 2 µM
1	1.8	1.9	7.7
2.6	2.8	2.7	18.4
1.5	1.1	1.3	4.4
2.6	2.6	2.4	24.8
1.6	1.4	1.7	7.9
2	3	2.5	12.7
**the effect of fentanyl alone: isolated platelets**			
c	F 2 ng/ml	F 2 µg/ml	ADP 2 µM
3	2.7	3.3	51.8
9.9	6.1	6.8	36.7
7.8	9	7.7	21.5
7.8	9	8.8	49.7
1.5	1.8	1.3	12.8
2.9	2.7	3.2	26.9
**the effect of fentanyl + ADP 2 µM: blood**			
c	F 2 ng/ml	F 2 µg/ml	adrenaline 10 µM
13	10.8	12	33.2
9.4	9.1	9.9	39.8
8.1	5.3	4.8	20.6
25	20.9	22.8	65.2
8.1	8.2	8.7	32.9
5.7	6.4	4.9	18.3
**the effect of fentanyl + ADP 2 µM: PRP**			
c	F 2 ng/ml	F 2 µg/ml	adrenaline 10 µM
10.2	8.9	10	36.8
18.7	20.4	18.7	58.6
4.4	4.9	4.2	28.2
36.7	27.2	28.9	71.8
9.8	9.8	9.3	47.7
13.2	13.9	14	41.8
**the effect of fentanyl + ADP 2 µM: isolated platelets**			
c	F 2 ng/ml	F 2 µg/ml	adrenaline 10 µM
44.1	42.6	43.9	70.2
51.7	50.5	45.8	76.8
23.1	24.7	21.1	41.8
52.7	51	48.9	80.8
14.4	13.3	11.7	41.7
26	27.5	23.8	46.3
**the effect of fentanyl (preincub.) + ADP 2 µM: blood**			
c	F 2 ng/ml	F 2 µg/ml	
17.8	17.5	18.1	
16.4	14.9	12.7	
5.8	4.2	7	
27	26.9	18.4	

11.2	9.9	9.5	
9.4	7.6	7.3	
**the effect of fentanyl (preincub.) + ADP 2 µM: PRP**			
c	F 2 ng/ml	F 2 µg/ml	
4.9	5.1	5	
8	7.4	8.2	
2.5	2.5	2.3	
19.1	17.3	16.9	
4.8	4.1	3.5	
6.4	6.1	7.3	
**the effect of fentanyl (preincub.) + ADP 2 µM: isolated platelets**			
c	F 2 ng/ml	F 2 µg/ml	
14.7	21.9	10.7	
35.9	25	22	
16.6	18.6	14.6	
36.6	32.9	28.8	
7.4	4	2.9	
7.1	6.9	5.9	
**the effect of fentanyl + ADP 5 µM + PM 1.3 µM: blood**			
c	PM	PM + F 2 ng/ml	PM + F 2 µg/ml
36.9	29.2	31.1	25.9
32.8	27.7	31.1	22.6
15.9	12.6	13.6	13.5
47.7	36.8	36.3	37.9
25.4	22.6	20.8	18
19.2	14.9	15.3	18
**the effect of fentanyl + ADP 5 µM + PM 1.3 µM: PRP**			
c	PM	PM + F 2 ng/ml	PM + F 2 µg/ml
44.4	34	37.2	34.9
43.5	33.9	33.6	33.4
28.8	21.2	20.4	19
56.3	42.3	44.2	44
30.6	24.5	22.3	22.9
40.6	27.9	28.4	27.2
**the effect of fentanyl + ADP 5 µM + PM 1.3 µM: isolated platelets**			
c	PM	PM + F 2 ng/ml	PM + F 2 µg/ml
35.7	3.6	3.6	3
42.2	9.6	8	9.6
21.8	2.4	2	2
46.1	7.3	9.8	9.5
7.8	1.2	0.9	0.9
8.1	2.3	2.4	2

SUPPLEMENTARY MATERIALS: interactions of fentanyl with plasma proteins

I. Molecular docking of fentanyl with human plasma proteins: the parameters of grid boxes

The sizes of the grid boxes with the grid box center coordinates were set as follows:

site-specific for Sudlow site 1 (2BXD) – xyz: 70; 62; 60 (center xyz: 1.736; –7.6; 5.6);

site-specific for Sudlow site 2 (2BXG) – xyz: 60; 90; 50 (center xyz: 4.591; –4.683; –15.990);

site-specific for drug site 3 (2VUE) – xyz: 64; 106; 74 (center xyz: 1.717; 9.943; 11.532);

blind for 3APU structure of α1-AGP – xyz: 50; 44; 48 (center xyz: 11.654; −0.085; 12.867);

blind for 3KQ0 structure of α1-AGP – xyz: 40; 42; 42 (center xyz: 21.198; −4.196; 2.146);

blind for 7OUB structure of α1-AGP – xyz: 46; 46; 42 (center xyz: −9.869; 33.081; −3.365);

site-specific for 3APU structure of α1-AGP – xyz: 50; 64; 60 (center xyz: 17.668; 3.316; 19.018);

site-specific for 3KQ0 structure of α1-AGP – xyz: 44; 50; 60 (center xyz: 29.124; −3.164; 2.146);

site-specific for 7OUB structure of α1-AGP – xyz: 62; 54; 52 (center xyz: −6.779; 30.241; 0.332);

blind for 1AV1 structure of ApoA-1 – xyz: 112; 114; 106 (center xyz: 46.061 ; 9.524; 25.064);

blind for 2N5E structure of ApoA-1 – xyz: 82; 56; 114 (center xyz: 11.133; 16.615; −55.867);

blind for 3R2P structure of ApoA-1 – xyz: 126; 84; 72 (center xyz: −13.601; 9.057; −19.200);

site-specific for 1AV1 structure of ApoA-1 – xyz: 40; 66; 40 (center xyz: 27.585; 17.365; −5.997);

site-specific for 2N5E structure of ApoA-1 – xyz: 58; 40; 40 (center xyz: 12.803; 11.190; −21.641);

site-specific for 3R2P structure of ApoA-1 – xyz: 58; 40; 52 (center xyz: −32.668; −2.369; −8.059).

II. The results of molecular docking simulations – raw data

1. Human Serum Albumin (HSA)

2BXD: site-specific docking – Sudlow site 1

```
LOWEST ENERGY DOCKED CONFORMATION
Estimated Free Energy of Binding    =   -8.28 kcal/mol  [=(1)+(2)+(3)-(4)]
Estimated Inhibition Constant, Ki   =  848.79 nM (nanomolar)  [Temperature = 298.15 K]

(1) Final Intermolecular Energy     =  -10.07 kcal/mol
    vdW + Hbond + desolv Energy     =   -9.87 kcal/mol
    Electrostatic Energy            =   -0.21 kcal/mol
(2) Final Total Internal Energy     =   -1.16 kcal/mol
(3) Torsional Free Energy           =   +1.79 kcal/mol
(4) Unbound System's Energy  [=(2)] =   -1.16 kcal/mol
```

Rank	Sub-Rank	Run	Binding Energy	Cluster RMSD	Reference RMSD	Grep Pattern
1	1	41	-8.28	0.00	13.86	RANKING
1	2	7	-7.99	1.56	13.24	RANKING
1	3	6	-7.90	1.33	13.31	RANKING
1	4	45	-7.33	1.68	12.81	RANKING
1	5	25	-6.97	1.71	13.87	RANKING
2	1	27	-7.92	0.00	10.41	RANKING
2	2	37	-7.91	0.08	10.43	RANKING
2	3	2	-7.81	0.21	10.34	RANKING
2	4	19	-7.50	1.90	10.49	RANKING
2	5	44	-6.91	1.73	10.40	RANKING
3	1	50	-7.85	0.00	12.86	RANKING
3	2	29	-7.58	0.78	12.98	RANKING
3	3	20	-7.54	1.02	13.34	RANKING
3	4	49	-7.46	0.74	13.02	RANKING
3	5	42	-7.41	1.44	12.71	RANKING
3	6	24	-7.38	1.12	13.29	RANKING
3	7	5	-7.36	1.18	12.89	RANKING
3	8	10	-7.29	1.35	12.44	RANKING
4	1	35	-7.71	0.00	11.47	RANKING
4	2	51	-7.70	1.21	12.07	RANKING
4	3	23	-7.69	0.05	11.46	RANKING
4	4	21	-7.69	0.29	11.35	RANKING
4	5	60	-7.65	0.61	11.37	RANKING
4	6	9	-7.61	0.53	11.37	RANKING
4	7	11	-7.59	0.44	11.36	RANKING
4	8	31	-7.43	1.43	12.21	RANKING
4	9	57	-7.41	1.24	12.14	RANKING
4	10	36	-7.30	0.89	11.61	RANKING
5	1	12	-7.51	0.00	10.01	RANKING
5	2	39	-7.02	1.22	9.75	RANKING
5	3	32	-6.97	1.93	10.48	RANKING
5	4	40	-6.95	2.00	10.44	RANKING
6	1	33	-7.43	0.00	9.99	RANKING
6	2	55	-7.10	1.02	9.91	RANKING
7	1	8	-7.28	0.00	13.22	RANKING
7	2	46	-6.86	0.86	13.03	RANKING
8	1	53	-7.20	0.00	10.45	RANKING
8	2	13	-7.18	0.22	10.45	RANKING
8	3	48	-7.03	1.96	10.48	RANKING
8	4	52	-6.57	1.94	10.44	RANKING
9	1	43	-7.11	0.00	11.84	RANKING
10	1	4	-7.00	0.00	10.55	RANKING
11	1	54	-6.79	0.00	7.31	RANKING
11	2	1	-6.76	0.32	7.42	RANKING
11	3	28	-6.66	1.05	7.59	RANKING
11	4	59	-6.64	0.53	7.59	RANKING
11	5	34	-6.36	0.87	7.76	RANKING
11	6	26	-6.20	0.72	7.36	RANKING
12	1	56	-6.62	0.00	8.98	RANKING
13	1	47	-6.60	0.00	7.74	RANKING
13	2	30	-6.60	0.31	7.62	RANKING
13	3	15	-6.16	1.98	7.25	RANKING
14	1	16	-6.51	0.00	7.17	RANKING

14	2	18	-6.43	1.95	7.74	RANKING
14	3	38	-6.21	1.87	8.31	RANKING
15	1	58	-6.43	0.00	6.95	RANKING
15	2	3	-6.26	1.55	7.44	RANKING
15	3	14	-6.20	1.57	7.43	RANKING
16	1	17	-6.42	0.00	7.99	RANKING
17	1	22	-6.03	0.00	6.91	RANKING

2BXG: site-specific docking – Sudlow site 2

```
LOWEST ENERGY DOCKED CONFORMATION
USER    Estimated Free Energy of Binding    =   -8.19 kcal/mol  [=(1)+(2)+(3)-(4)]
USER    Estimated Inhibition Constant, Ki   =  997.13 nM (nanomolar)  [Temperature = 298.15 K]
USER
USER    (1) Final Intermolecular Energy     =   -9.98 kcal/mol
USER        vdW + Hbond + desolv Energy     =   -9.85 kcal/mol
USER        Electrostatic Energy            =   -0.13 kcal/mol
USER    (2) Final Total Internal Energy     =   -1.28 kcal/mol
USER    (3) Torsional Free Energy           =   +1.79 kcal/mol
USER    (4) Unbound System's Energy  [=(2)] =   -1.28 kcal/mol
```

Rank	Sub-Rank	Run	Binding Energy	Cluster RMSD	Reference RMSD	Grep Pattern
1	1	28	-6.94	0.00	18.65	RANKING
1	2	1	-6.81	1.70	19.21	RANKING
1	3	7	-6.77	1.11	19.38	RANKING
1	4	52	-6.67	1.37	19.86	RANKING
1	5	5	-6.56	1.11	19.35	RANKING
1	6	26	-6.51	1.76	20.52	RANKING
1	7	53	-6.22	1.93	19.50	RANKING
1	8	45	-6.13	1.95	19.57	RANKING
2	1	30	-6.75	0.00	22.22	RANKING
2	2	60	-6.24	1.82	22.28	RANKING
2	3	34	-5.86	1.51	22.13	RANKING
3	1	13	-6.53	0.00	23.18	RANKING
3	2	20	-5.55	1.28	23.08	RANKING
4	1	17	-6.50	0.00	20.25	RANKING
4	2	23	-6.46	1.04	20.01	RANKING
4	3	19	-6.00	1.29	19.21	RANKING
5	1	27	-6.43	0.00	22.76	RANKING
5	2	14	-6.25	1.20	22.47	RANKING
5	3	49	-6.19	1.40	22.06	RANKING
5	4	36	-6.12	1.12	22.86	RANKING
6	1	22	-6.43	0.00	16.65	RANKING
7	1	51	-6.29	0.00	21.03	RANKING
8	1	4	-6.27	0.00	22.45	RANKING
9	1	21	-6.07	0.00	28.01	RANKING
9	2	43	-5.94	1.34	28.48	RANKING
10	1	11	-6.06	0.00	21.11	RANKING
11	1	58	-6.03	0.00	23.31	RANKING
12	1	59	-6.01	0.00	19.59	RANKING
12	2	24	-5.88	1.06	20.56	RANKING
13	1	3	-5.96	0.00	27.74	RANKING
14	1	41	-5.92	0.00	21.88	RANKING
14	2	10	-5.91	1.30	22.55	RANKING
15	1	50	-5.88	0.00	8.65	RANKING
16	1	16	-5.85	0.00	21.91	RANKING
17	1	56	-5.83	0.00	20.37	RANKING
17	2	33	-5.78	1.83	20.90	RANKING
17	3	9	-5.56	0.98	19.94	RANKING
18	1	32	-5.80	0.00	22.29	RANKING
19	1	40	-5.79	0.00	13.61	RANKING
19	2	57	-5.67	1.94	13.34	RANKING
19	3	42	-5.44	1.87	13.79	RANKING
20	1	39	-5.72	0.00	20.11	RANKING
21	1	2	-5.71	0.00	20.77	RANKING
21	2	18	-5.29	1.27	20.67	RANKING
22	1	37	-5.63	0.00	25.51	RANKING

23	1	47	-5.59	0.00	21.55	RANKING
24	1	6	-5.51	0.00	6.78	RANKING
25	1	54	-5.50	0.00	11.99	RANKING
26	1	12	-5.39	0.00	20.82	RANKING
27	1	46	-5.35	0.00	13.04	RANKING
28	1	25	-5.29	0.00	12.49	RANKING
29	1	15	-5.24	0.00	10.77	RANKING
30	1	48	-5.14	0.00	31.41	RANKING
31	1	8	-5.07	0.00	17.78	RANKING
32	1	35	-5.06	0.00	12.07	RANKING
33	1	55	-5.03	0.00	31.20	RANKING
34	1	38	-5.01	0.00	13.55	RANKING
35	1	29	-4.80	0.00	21.76	RANKING
36	1	44	-4.75	0.00	12.43	RANKING
37	1	31	-4.71	0.00	19.39	RANKING

2VUE: site-specific docking – drug site 3

```
LOWEST ENERGY DOCKED CONFORMATION
USER    Estimated Free Energy of Binding    =   -9.21 kcal/mol  [=(1)+(2)+(3)-(4)]
USER    Estimated Inhibition Constant, Ki   =  178.74 nM (nanomolar)  [Temperature = 298.15 K]
USER
USER    (1) Final Intermolecular Energy     =  -11.00 kcal/mol
USER        vdW + Hbond + desolv Energy     =  -11.14 kcal/mol
USER        Electrostatic Energy            =   +0.14 kcal/mol
USER    (2) Final Total Internal Energy     =   -0.83 kcal/mol
USER    (3) Torsional Free Energy           =   +1.79 kcal/mol
USER    (4) Unbound System's Energy  [=(2)] =   -0.83 kcal/mol
```

Rank	Sub-Rank	Run	Binding Energy	Cluster RMSD	Reference RMSD	Grep Pattern
1	1	58	-9.21	0.00	17.31	RANKING
1	2	42	-9.10	0.22	17.31	RANKING
1	3	50	-8.70	0.70	17.18	RANKING
1	4	1	-8.63	1.01	16.66	RANKING
1	5	51	-8.55	1.94	17.79	RANKING
1	6	10	-8.54	1.00	16.80	RANKING
1	7	41	-8.51	0.89	17.61	RANKING
1	8	52	-8.25	0.86	17.36	RANKING
1	9	59	-7.64	1.79	18.78	RANKING
2	1	46	-9.02	0.00	16.98	RANKING
2	2	57	-8.98	0.73	17.00	RANKING
2	3	25	-8.91	0.85	17.34	RANKING
2	4	40	-8.87	1.34	17.31	RANKING
2	5	31	-8.73	1.37	17.65	RANKING
2	6	36	-8.57	1.35	17.71	RANKING
2	7	17	-8.56	1.38	17.45	RANKING
2	8	30	-8.53	1.43	17.87	RANKING
2	9	28	-8.48	1.29	17.16	RANKING
2	10	53	-8.35	0.82	16.87	RANKING
2	11	7	-8.29	0.97	17.55	RANKING
2	12	23	-8.24	1.43	17.00	RANKING
2	13	37	-8.23	1.61	17.53	RANKING
3	1	3	-8.98	0.00	14.38	RANKING
3	2	14	-8.67	1.40	15.97	RANKING
3	3	55	-8.57	1.05	15.05	RANKING
3	4	5	-8.57	0.85	14.65	RANKING
3	5	54	-8.30	1.41	15.28	RANKING
3	6	6	-8.30	0.70	14.24	RANKING
3	7	22	-7.87	1.74	15.46	RANKING
4	1	8	-8.87	0.00	14.11	RANKING
5	1	39	-8.27	0.00	7.66	RANKING
5	2	2	-7.08	1.02	7.50	RANKING
5	3	29	-6.86	1.58	8.21	RANKING
5	4	18	-6.18	1.77	7.25	RANKING
6	1	4	-8.09	0.00	15.04	RANKING
6	2	34	-7.90	0.94	14.77	RANKING
7	1	44	-8.03	0.00	7.36	RANKING

7	2	13	-6.81	1.39	7.60	RANKING
7	3	26	-6.44	1.97	7.72	RANKING
7	4	19	-6.30	1.98	7.58	RANKING
8	1	35	-7.90	0.00	21.01	RANKING
8	2	48	-7.82	1.75	19.46	RANKING
8	3	32	-7.75	1.33	19.89	RANKING
9	1	49	-7.89	0.00	19.18	RANKING
9	2	21	-7.38	1.17	19.57	RANKING
10	1	15	-7.76	0.00	12.65	RANKING
11	1	9	-7.75	0.00	21.10	RANKING
11	2	12	-7.62	1.36	20.67	RANKING
11	3	45	-7.55	1.40	20.64	RANKING
12	1	27	-7.74	0.00	21.81	RANKING
12	2	60	-7.26	0.64	21.75	RANKING
13	1	56	-7.73	0.00	21.84	RANKING
13	2	38	-7.11	1.17	21.59	RANKING
14	1	11	-7.26	0.00	20.60	RANKING
14	2	33	-6.55	1.56	20.51	RANKING
15	1	20	-6.63	0.00	15.98	RANKING
15	2	43	-6.57	0.36	15.75	RANKING
16	1	24	-6.55	0.00	8.17	RANKING
17	1	16	-6.21	0.00	15.37	RANKING
18	1	47	-5.77	0.00	8.31	RANKING

2. α1-Acid Glycoprotein (α1-AGP)

3APU: blind docking

```
LOWEST ENERGY DOCKED CONFORMATION
USER    Estimated Free Energy of Binding    =   -6.86 kcal/mol  [=(1)+(2)+(3)-(4)]
USER    Estimated Inhibition Constant, Ki   =    9.31 uM (micromolar)  [Temperature = 298.15 K]
USER
USER    (1) Final Intermolecular Energy     =   -8.65 kcal/mol
USER        vdW + Hbond + desolv Energy     =   -8.14 kcal/mol
USER        Electrostatic Energy            =   -0.52 kcal/mol
USER    (2) Final Total Internal Energy     =   -1.62 kcal/mol
USER    (3) Torsional Free Energy           =   +1.79 kcal/mol
USER    (4) Unbound System's Energy  [=(2)] =   -1.62 kcal/mol
```

Rank	Sub-Rank	Run	Binding Energy	Cluster RMSD	Reference RMSD	Grep Pattern
1	1	38	-6.86	0.00	21.62	RANKING
1	2	13	-6.48	1.71	22.89	RANKING
1	3	12	-6.47	1.48	22.57	RANKING
1	4	21	-6.47	1.26	22.14	RANKING
2	1	10	-6.75	0.00	23.39	RANKING
2	2	6	-6.22	1.96	22.75	RANKING
3	1	59	-5.86	0.00	23.92	RANKING
3	2	41	-5.50	0.97	23.47	RANKING
4	1	22	-5.71	0.00	21.87	RANKING
5	1	27	-5.27	0.00	21.28	RANKING
6	1	50	-5.20	0.00	24.65	RANKING
6	2	48	-5.18	1.60	24.80	RANKING
7	1	28	-4.87	0.00	20.23	RANKING
7	2	57	-4.79	1.48	19.26	RANKING
8	1	37	-4.69	0.00	16.70	RANKING
8	2	2	-4.48	1.60	17.02	RANKING
9	1	53	-4.64	0.00	22.14	RANKING
9	2	19	-4.64	0.17	22.16	RANKING
9	3	11	-4.63	1.83	22.08	RANKING
9	4	58	-4.46	1.39	21.73	RANKING
9	5	43	-4.46	0.35	22.20	RANKING
9	6	32	-4.38	1.00	22.17	RANKING
9	7	34	-4.32	0.99	22.28	RANKING
10	1	29	-4.62	0.00	23.12	RANKING
11	1	15	-4.61	0.00	28.23	RANKING
12	1	23	-4.58	0.00	10.61	RANKING

13	1	25	-4.57	0.00	23.09	RANKING
14	1	45	-4.54	0.00	20.99	RANKING
15	1	47	-4.41	0.00	20.76	RANKING
15	2	26	-4.34	1.83	21.04	RANKING
15	3	24	-3.98	1.86	20.96	RANKING
16	1	9	-4.39	0.00	24.73	RANKING
16	2	3	-3.92	1.41	24.46	RANKING
17	1	16	-4.39	0.00	23.54	RANKING
18	1	42	-4.38	0.00	21.90	RANKING
19	1	49	-4.37	0.00	18.82	RANKING
20	1	18	-4.33	0.00	11.68	RANKING
21	1	7	-4.26	0.00	12.47	RANKING
22	1	46	-4.16	0.00	22.09	RANKING
22	2	52	-4.04	1.73	21.87	RANKING
23	1	30	-4.05	0.00	21.89	RANKING
24	1	54	-4.05	0.00	22.89	RANKING
25	1	39	-4.00	0.00	11.73	RANKING
25	2	14	-4.00	1.95	10.11	RANKING
26	1	20	-3.99	0.00	19.22	RANKING
27	1	55	-3.96	0.00	20.73	RANKING
27	2	31	-3.87	0.39	20.71	RANKING
27	3	17	-3.86	0.25	20.73	RANKING
28	1	4	-3.94	0.00	27.69	RANKING
29	1	35	-3.92	0.00	22.23	RANKING
30	1	36	-3.90	0.00	26.45	RANKING
31	1	56	-3.89	0.00	18.30	RANKING
32	1	44	-3.85	0.00	21.19	RANKING
33	1	33	-3.79	0.00	10.29	RANKING
33	2	5	-3.75	1.01	10.70	RANKING
34	1	1	-3.77	0.00	7.69	RANKING
35	1	40	-3.75	0.00	21.07	RANKING
36	1	8	-3.74	0.00	9.58	RANKING
37	1	51	-3.73	0.00	23.12	RANKING
38	1	60	-3.41	0.00	10.03	RANKING

3APU: site-specific docking

```
LOWEST ENERGY DOCKED CONFORMATION
USER    Estimated Free Energy of Binding    =   -8.43 kcal/mol  [=(1)+(2)+(3)-(4)]
USER    Estimated Inhibition Constant, Ki   =  666.84 nM (nanomolar)  [Temperature = 298.15 K]
USER
USER    (1) Final Intermolecular Energy     =  -10.22 kcal/mol
USER        vdW + Hbond + desolv Energy     =   -9.87 kcal/mol
USER        Electrostatic Energy            =   -0.34 kcal/mol
USER    (2) Final Total Internal Energy     =   -1.64 kcal/mol
USER    (3) Torsional Free Energy           =   +1.79 kcal/mol
USER    (4) Unbound System's Energy  [=(2)] =   -1.64 kcal/mol
```

Rank	Sub-Rank	Run	Binding Energy	Cluster RMSD	Reference RMSD	Grep Pattern
1	1	34	-8.43	0.00	20.01	RANKING
1	2	14	-8.38	0.15	20.01	RANKING
1	3	50	-8.18	0.24	20.05	RANKING
2	1	56	-8.24	0.00	19.68	RANKING
2	2	60	-8.23	0.27	19.69	RANKING
2	3	20	-8.22	0.17	19.62	RANKING
2	4	16	-8.02	0.36	19.70	RANKING
2	5	24	-7.99	0.79	19.73	RANKING
2	6	13	-7.91	0.48	19.63	RANKING
2	7	26	-7.87	0.76	19.67	RANKING
2	8	3	-7.84	0.62	19.64	RANKING
2	9	41	-7.82	0.66	19.59	RANKING
2	10	42	-7.80	0.62	19.64	RANKING
2	11	17	-7.77	0.92	19.63	RANKING
2	12	12	-7.66	1.06	19.65	RANKING
3	1	28	-7.94	0.00	20.35	RANKING
3	2	2	-7.90	0.54	20.45	RANKING
3	3	39	-7.73	0.56	20.38	RANKING

3	4	47	-7.72	0.89	20.74	RANKING
3	5	33	-7.67	0.73	20.62	RANKING
4	1	43	-7.93	0.00	20.87	RANKING
5	1	7	-7.92	0.00	20.00	RANKING
6	1	30	-7.88	0.00	22.19	RANKING
6	2	59	-7.83	0.37	21.89	RANKING
6	3	5	-7.81	1.10	21.50	RANKING
6	4	6	-7.80	1.05	21.60	RANKING
6	5	31	-7.79	1.00	21.66	RANKING
6	6	11	-7.78	1.07	21.61	RANKING
6	7	54	-7.77	0.36	21.94	RANKING
6	8	55	-7.77	0.94	21.76	RANKING
6	9	40	-7.77	0.29	22.07	RANKING
6	10	15	-7.77	1.06	21.57	RANKING
6	11	38	-7.76	0.95	21.71	RANKING
6	12	19	-7.75	0.98	21.63	RANKING
6	13	48	-7.74	0.96	21.66	RANKING
6	14	58	-7.74	0.97	21.67	RANKING
6	15	35	-7.68	1.89	21.89	RANKING
6	16	10	-7.67	0.95	21.68	RANKING
6	17	37	-7.60	1.85	21.91	RANKING
6	18	52	-7.58	1.85	21.96	RANKING
6	19	51	-7.58	1.94	21.83	RANKING
6	20	8	-7.57	1.86	21.87	RANKING
6	21	49	-7.55	1.36	22.52	RANKING
6	22	23	-7.55	1.40	22.52	RANKING
6	23	53	-7.54	1.94	21.82	RANKING
6	24	29	-7.53	1.33	22.56	RANKING
6	25	22	-7.52	1.79	21.97	RANKING
6	26	45	-7.52	1.85	21.97	RANKING
6	27	18	-7.52	1.00	21.60	RANKING
6	28	32	-7.52	1.81	21.98	RANKING
6	29	36	-7.50	1.79	22.02	RANKING
6	30	1	-7.50	1.77	22.07	RANKING
6	31	57	-7.49	1.23	22.53	RANKING
6	32	44	-7.49	1.24	22.51	RANKING
6	33	46	-7.48	1.77	21.96	RANKING
7	1	21	-7.86	0.00	20.63	RANKING
8	1	9	-7.62	0.00	20.19	RANKING
9	1	25	-7.50	0.00	20.30	RANKING
9	2	27	-7.45	1.94	20.19	RANKING
9	3	4	-7.43	1.60	20.51	RANKING

3KQ0: blind docking

```
LOWEST ENERGY DOCKED CONFORMATION
USER    Estimated Free Energy of Binding    =   -6.41 kcal/mol  [=(1)+(2)+(3)-(4)]
USER    Estimated Inhibition Constant, Ki   =   20.16 uM (micromolar)  [Temperature = 298.15 K]
USER
USER    (1) Final Intermolecular Energy     =   -8.20 kcal/mol
USER        vdW + Hbond + desolv Energy     =   -8.06 kcal/mol
USER        Electrostatic Energy            =   -0.14 kcal/mol
USER    (2) Final Total Internal Energy     =   -1.36 kcal/mol
USER    (3) Torsional Free Energy           =   +1.79 kcal/mol
USER    (4) Unbound System's Energy  [=(2)] =   -1.36 kcal/mol
```

Rank	Sub-Rank	Run	Binding Energy	Cluster RMSD	Reference RMSD	Grep Pattern
1	1	43	-6.41	0.00	24.98	RANKING
2	1	1	-6.31	0.00	23.66	RANKING
2	2	23	-6.14	1.74	23.45	RANKING
3	1	41	-6.28	0.00	24.75	RANKING
3	2	27	-6.23	0.41	24.65	RANKING
3	3	44	-5.96	1.95	24.66	RANKING
4	1	37	-6.20	0.00	23.93	RANKING
4	2	50	-6.00	0.47	23.76	RANKING
4	3	29	-5.98	0.85	23.38	RANKING
4	4	59	-5.93	1.03	24.25	RANKING

4	5	11	-5.93	1.04	24.08	RANKING
4	6	57	-5.93	1.55	24.05	RANKING
4	7	53	-5.90	1.03	24.25	RANKING
4	8	2	-5.84	1.48	24.15	RANKING
4	9	26	-5.46	0.96	24.04	RANKING
5	1	38	-6.16	0.00	24.17	RANKING
5	2	48	-5.83	1.23	23.58	RANKING
5	3	5	-5.63	1.67	24.01	RANKING
6	1	55	-6.05	0.00	22.53	RANKING
7	1	7	-5.75	0.00	23.49	RANKING
8	1	33	-5.59	0.00	26.99	RANKING
8	2	49	-5.55	0.21	27.05	RANKING
8	3	15	-5.15	0.42	27.07	RANKING
8	4	45	-4.91	1.19	27.32	RANKING
8	5	17	-4.85	1.23	27.54	RANKING
9	1	19	-5.46	0.00	27.65	RANKING
9	2	42	-4.97	1.22	28.17	RANKING
9	3	21	-4.77	1.46	28.54	RANKING
10	1	24	-5.37	0.00	28.13	RANKING
10	2	56	-5.31	0.64	28.07	RANKING
10	3	20	-5.06	1.96	28.07	RANKING
11	1	40	-5.32	0.00	27.43	RANKING
11	2	60	-4.78	1.37	27.44	RANKING
12	1	22	-5.28	0.00	27.83	RANKING
12	2	35	-5.04	0.97	28.08	RANKING
13	1	18	-5.17	0.00	27.94	RANKING
14	1	13	-5.15	0.00	15.54	RANKING
15	1	32	-5.11	0.00	24.38	RANKING
15	2	25	-4.93	0.42	24.38	RANKING
16	1	4	-5.11	0.00	18.54	RANKING
17	1	58	-4.97	0.00	27.30	RANKING
18	1	6	-4.95	0.00	14.85	RANKING
19	1	36	-4.92	0.00	21.15	RANKING
19	2	31	-4.82	0.39	21.06	RANKING
19	3	51	-4.59	1.95	20.89	RANKING
19	4	10	-4.48	1.90	22.39	RANKING
19	5	28	-4.45	1.81	22.18	RANKING
19	6	14	-4.42	1.93	20.70	RANKING
20	1	52	-4.90	0.00	28.48	RANKING
21	1	9	-4.89	0.00	17.62	RANKING
22	1	54	-4.78	0.00	19.96	RANKING
22	2	47	-4.11	1.81	20.17	RANKING
23	1	12	-4.75	0.00	21.55	RANKING
23	2	16	-4.52	1.29	21.86	RANKING
23	3	3	-4.39	1.76	22.20	RANKING
24	1	39	-4.70	0.00	28.84	RANKING
25	1	34	-4.66	0.00	30.02	RANKING
26	1	8	-4.31	0.00	28.05	RANKING
27	1	30	-4.26	0.00	29.26	RANKING
28	1	46	-4.23	0.00	17.28	RANKING

3KQ0: site-specific docking

```
LOWEST ENERGY DOCKED CONFORMATION
USER    Estimated Free Energy of Binding    =   -8.63 kcal/mol  [=(1)+(2)+(3)-(4)]
USER    Estimated Inhibition Constant, Ki   =  473.77 nM (nanomolar)  [Temperature = 298.15 K]
USER
USER    (1) Final Intermolecular Energy     =  -10.42 kcal/mol
USER        vdW + Hbond + desolv Energy     =  -10.24 kcal/mol
USER        Electrostatic Energy            =   -0.18 kcal/mol
USER    (2) Final Total Internal Energy     =   -0.83 kcal/mol
USER    (3) Torsional Free Energy           =   +1.79 kcal/mol
USER    (4) Unbound System's Energy  [=(2)] =   -0.83 kcal/mol
```

Rank	Sub-Rank	Run	Binding Energy	Cluster RMSD	Reference RMSD	Grep Pattern
1	1	29	-8.63	0.00	24.07	RANKING
2	1	24	-8.32	0.00	25.19	RANKING

2	2	22	-8.27	1.81	23.87	RANKING
2	3	44	-8.21	1.81	24.17	RANKING
2	4	35	-8.18	0.13	25.15	RANKING
2	5	21	-7.85	2.00	23.76	RANKING
2	6	10	-7.48	1.84	24.52	RANKING
3	1	34	-8.22	0.00	21.23	RANKING
4	1	16	-8.20	0.00	25.28	RANKING
4	2	9	-8.12	0.20	25.28	RANKING
4	3	46	-8.11	0.31	25.32	RANKING
4	4	30	-8.11	0.25	25.26	RANKING
4	5	48	-8.07	0.27	25.22	RANKING
4	6	6	-7.71	1.46	24.85	RANKING
4	7	7	-7.69	1.38	25.02	RANKING
4	8	33	-7.68	1.40	24.96	RANKING
4	9	25	-7.66	1.37	24.97	RANKING
4	10	49	-7.64	1.45	24.95	RANKING
4	11	43	-7.62	1.31	24.68	RANKING
4	12	13	-7.58	1.36	24.68	RANKING
4	13	27	-7.58	1.39	25.00	RANKING
4	14	57	-7.57	1.34	24.61	RANKING
4	15	58	-7.54	1.38	24.70	RANKING
4	16	36	-7.45	1.36	24.77	RANKING
4	17	56	-7.37	1.36	24.76	RANKING
5	1	23	-8.18	0.00	21.35	RANKING
5	2	4	-7.90	1.71	21.64	RANKING
5	3	53	-7.89	1.74	21.67	RANKING
5	4	5	-7.85	1.73	21.59	RANKING
5	5	42	-7.85	1.73	21.65	RANKING
5	6	32	-7.84	1.71	21.67	RANKING
5	7	47	-7.80	1.72	21.54	RANKING
5	8	31	-7.53	1.76	21.85	RANKING
6	1	51	-8.06	0.00	23.17	RANKING
6	2	15	-8.02	0.36	23.09	RANKING
6	3	11	-7.96	0.49	23.16	RANKING
6	4	20	-7.93	0.79	23.49	RANKING
6	5	38	-7.89	0.50	23.22	RANKING
6	6	52	-7.55	1.15	23.78	RANKING
6	7	18	-7.36	1.84	23.53	RANKING
7	1	28	-8.00	0.00	27.01	RANKING
8	1	1	-7.86	0.00	24.26	RANKING
8	2	39	-7.84	0.47	24.03	RANKING
8	3	17	-7.83	0.49	24.01	RANKING
8	4	41	-7.15	1.88	24.75	RANKING
9	1	8	-7.85	0.00	24.59	RANKING
9	2	60	-7.83	0.18	24.69	RANKING
9	3	19	-7.74	0.36	24.83	RANKING
9	4	14	-7.73	0.40	24.87	RANKING
9	5	50	-7.72	0.25	24.75	RANKING
9	6	37	-7.72	0.20	24.68	RANKING
9	7	26	-7.70	0.27	24.47	RANKING
9	8	59	-7.57	0.44	24.87	RANKING
9	9	54	-7.52	0.57	24.40	RANKING
10	1	55	-7.74	0.00	24.55	RANKING
10	2	40	-7.47	1.19	24.23	RANKING
11	1	3	-7.36	0.00	20.84	RANKING
11	2	2	-7.24	0.85	21.00	RANKING
12	1	45	-7.29	0.00	22.36	RANKING
13	1	12	-7.13	0.00	22.58	RANKING

7OUB: blind docking

```
LOWEST ENERGY DOCKED CONFORMATION
USER    Estimated Free Energy of Binding    =   -6.97 kcal/mol  [=(1)+(2)+(3)-(4)]
USER    Estimated Inhibition Constant, Ki   =    7.72 uM (micromolar)  [Temperature = 298.15 K]
USER
USER    (1) Final Intermolecular Energy     =   -8.76 kcal/mol
USER        vdW + Hbond + desolv Energy     =   -7.83 kcal/mol
USER        Electrostatic Energy            =   -0.94 kcal/mol
USER    (2) Final Total Internal Energy     =   -1.22 kcal/mol
USER    (3) Torsional Free Energy           =   +1.79 kcal/mol
USER    (4) Unbound System's Energy  [=(2)] =   -1.22 kcal/mol
```

Rank	Sub-Rank	Run	Binding Energy	Cluster RMSD	Reference RMSD	Grep Pattern
1	1	25	-6.97	0.00	29.17	RANKING
1	2	29	-6.78	1.17	29.77	RANKING
1	3	42	-6.76	0.60	29.46	RANKING
1	4	12	-6.74	1.65	30.35	RANKING
1	5	2	-6.53	1.74	29.44	RANKING
1	6	17	-6.52	0.75	29.51	RANKING
1	7	38	-5.90	1.74	29.52	RANKING
2	1	9	-6.78	0.00	30.65	RANKING
2	2	19	-6.63	1.05	30.58	RANKING
2	3	59	-6.58	1.09	30.61	RANKING
2	4	16	-6.43	1.31	30.48	RANKING
2	5	41	-6.33	0.60	30.80	RANKING
2	6	40	-6.28	1.25	30.41	RANKING
2	7	48	-6.27	0.60	30.53	RANKING
3	1	4	-6.67	0.00	30.13	RANKING
3	2	8	-6.23	0.82	30.17	RANKING
4	1	26	-6.63	0.00	32.06	RANKING
4	2	13	-6.57	1.95	30.64	RANKING
4	3	14	-6.46	1.80	30.70	RANKING
4	4	20	-6.45	1.92	30.58	RANKING
4	5	37	-6.28	0.86	32.04	RANKING
5	1	6	-6.47	0.00	31.91	RANKING
5	2	60	-5.90	1.26	32.09	RANKING
6	1	33	-6.22	0.00	29.15	RANKING
7	1	53	-6.11	0.00	31.40	RANKING
7	2	18	-5.92	1.00	31.20	RANKING
7	3	23	-5.52	1.73	30.66	RANKING
8	1	43	-6.05	0.00	27.49	RANKING
8	2	24	-5.31	1.07	27.69	RANKING
8	3	47	-5.23	1.02	27.74	RANKING
9	1	55	-6.05	0.00	29.00	RANKING
10	1	30	-5.96	0.00	30.62	RANKING
11	1	46	-5.94	0.00	30.01	RANKING
12	1	7	-5.93	0.00	30.45	RANKING
12	2	50	-5.66	1.96	30.97	RANKING
13	1	36	-5.84	0.00	31.54	RANKING
14	1	49	-5.75	0.00	29.46	RANKING
15	1	15	-5.65	0.00	30.08	RANKING
15	2	51	-5.49	1.42	30.36	RANKING
15	3	52	-5.42	1.41	29.44	RANKING
16	1	3	-5.54	0.00	32.06	RANKING
17	1	10	-5.51	0.00	30.94	RANKING
18	1	22	-5.43	0.00	30.99	RANKING
19	1	34	-5.17	0.00	31.94	RANKING
20	1	57	-4.68	0.00	47.72	RANKING
21	1	5	-4.57	0.00	20.48	RANKING
22	1	21	-4.52	0.00	47.45	RANKING
23	1	11	-4.46	0.00	50.14	RANKING
24	1	45	-4.42	0.00	49.47	RANKING
25	1	44	-4.15	0.00	48.68	RANKING
26	1	1	-4.09	0.00	29.83	RANKING
27	1	32	-4.02	0.00	48.52	RANKING
27	2	35	-3.82	1.06	48.85	RANKING
28	1	58	-4.01	0.00	48.68	RANKING
29	1	27	-4.00	0.00	34.42	RANKING
30	1	39	-3.90	0.00	48.33	RANKING
31	1	56	-3.81	0.00	28.88	RANKING
32	1	28	-3.75	0.00	47.49	RANKING
33	1	54	-3.57	0.00	27.83	RANKING
34	1	31	-3.52	0.00	45.61	RANKING

7OUB: site-specific docking

```
LOWEST ENERGY DOCKED CONFORMATION
USER    Estimated Free Energy of Binding    =   -8.19 kcal/mol  [=(1)+(2)+(3)-(4)]
```

```
USER    Estimated Inhibition Constant, Ki   =  995.26 nM (nanomolar)  [Temperature = 298.15 K]
USER
USER    (1) Final Intermolecular Energy     =   -9.98 kcal/mol
USER        vdW + Hbond + desolv Energy     =   -9.13 kcal/mol
USER        Electrostatic Energy            =   -0.85 kcal/mol
USER    (2) Final Total Internal Energy     =   -1.31 kcal/mol
USER    (3) Torsional Free Energy           =   +1.79 kcal/mol
USER    (4) Unbound System's Energy  [=(2)] =   -1.31 kcal/mol
```

Rank	Sub-Rank	Run	Binding Energy	Cluster RMSD	Reference RMSD	Grep Pattern
1	1	60	-8.19	0.00	28.52	RANKING
2	1	58	-8.03	0.00	30.12	RANKING
2	2	29	-8.02	1.36	30.25	RANKING
2	3	43	-8.01	0.99	30.42	RANKING
2	4	22	-7.93	1.05	30.52	RANKING
2	5	2	-7.77	0.91	30.30	RANKING
2	6	52	-7.71	1.41	30.36	RANKING
3	1	44	-7.95	0.00	29.57	RANKING
4	1	47	-7.94	0.00	28.44	RANKING
4	2	46	-7.92	0.36	28.33	RANKING
4	3	34	-7.92	0.26	28.37	RANKING
4	4	26	-7.90	0.19	28.45	RANKING
4	5	24	-7.90	0.39	28.32	RANKING
4	6	40	-7.90	0.40	28.36	RANKING
4	7	39	-7.90	0.45	28.33	RANKING
4	8	5	-7.89	0.28	28.41	RANKING
4	9	57	-7.88	0.39	28.33	RANKING
4	10	53	-7.88	0.26	28.42	RANKING
4	11	3	-7.88	0.22	28.44	RANKING
4	12	23	-7.87	0.23	28.44	RANKING
4	13	54	-7.86	0.29	28.41	RANKING
4	14	11	-7.86	0.38	28.44	RANKING
4	15	27	-7.85	0.29	28.40	RANKING
4	16	33	-7.85	0.37	28.43	RANKING
4	17	20	-7.85	0.35	28.46	RANKING
4	18	15	-7.85	0.31	28.46	RANKING
4	19	41	-7.85	0.38	28.45	RANKING
4	20	16	-7.85	0.39	28.48	RANKING
4	21	13	-7.85	0.34	28.43	RANKING
4	22	17	-7.84	0.39	28.42	RANKING
4	23	19	-7.84	0.46	28.35	RANKING
4	24	28	-7.84	0.34	28.50	RANKING
4	25	18	-7.84	0.36	28.43	RANKING
4	26	8	-7.84	0.41	28.46	RANKING
4	27	4	-7.83	0.53	28.30	RANKING
4	28	25	-7.83	0.30	28.39	RANKING
4	29	14	-7.83	0.43	28.34	RANKING
4	30	37	-7.83	0.38	28.48	RANKING
4	31	32	-7.83	0.38	28.49	RANKING
4	32	48	-7.83	0.35	28.48	RANKING
4	33	38	-7.83	0.49	28.42	RANKING
4	34	9	-7.83	0.31	28.44	RANKING
4	35	59	-7.82	0.50	28.29	RANKING
4	36	42	-7.82	0.35	28.47	RANKING
4	37	35	-7.82	0.36	28.48	RANKING
4	38	21	-7.82	0.32	28.47	RANKING
4	39	45	-7.81	0.30	28.49	RANKING
4	40	7	-7.81	0.44	28.35	RANKING
4	41	30	-7.81	0.34	28.47	RANKING
4	42	6	-7.80	0.44	28.44	RANKING
4	43	10	-7.80	0.49	28.33	RANKING
4	44	31	-7.80	0.37	28.48	RANKING
4	45	50	-7.79	0.42	28.48	RANKING
4	46	51	-7.79	0.39	28.49	RANKING
4	47	55	-7.78	0.46	28.40	RANKING
5	1	49	-7.51	0.00	30.37	RANKING
5	2	12	-7.43	0.29	30.53	RANKING
5	3	1	-7.37	0.72	30.47	RANKING
6	1	56	-7.50	0.00	32.21	RANKING
6	2	36	-7.40	1.86	31.50	RANKING

3. Apolipoprotein A-1 (ApoA-1)

1AV1: blind docking

```
LOWEST ENERGY DOCKED CONFORMATION
USER    Estimated Free Energy of Binding    =   -4.88 kcal/mol  [=(1)+(2)+(3)-(4)]
USER    Estimated Inhibition Constant, Ki   =  263.56 uM (micromolar)  [Temperature = 298.15 K]
USER
USER    (1) Final Intermolecular Energy     =   -6.67 kcal/mol
USER        vdW + Hbond + desolv Energy     =   -5.83 kcal/mol
USER        Electrostatic Energy            =   -0.85 kcal/mol
USER    (2) Final Total Internal Energy     =   -1.22 kcal/mol
USER    (3) Torsional Free Energy           =   +1.79 kcal/mol
USER    (4) Unbound System's Energy  [=(2)] =   -1.22 kcal/mol
```

Rank	Sub-Rank	Run	Binding Energy	Cluster RMSD	Reference RMSD	Grep Pattern
1	1	12	-4.88	0.00	32.51	RANKING
1	2	13	-4.87	0.34	32.55	RANKING
1	3	26	-4.02	1.03	33.04	RANKING
2	1	51	-4.80	0.00	27.26	RANKING
2	2	9	-4.08	1.23	26.45	RANKING
3	1	46	-4.60	0.00	28.71	RANKING
3	2	57	-4.34	0.33	28.74	RANKING
3	3	24	-4.33	0.71	28.52	RANKING
3	4	28	-3.77	1.25	29.69	RANKING
4	1	54	-4.42	0.00	27.78	RANKING
4	2	39	-4.30	0.27	27.73	RANKING
5	1	25	-4.36	0.00	26.37	RANKING
6	1	53	-4.33	0.00	28.28	RANKING
7	1	22	-4.20	0.00	31.52	RANKING
7	2	44	-4.17	0.86	32.06	RANKING
8	1	42	-4.03	0.00	25.09	RANKING
8	2	55	-3.56	1.85	24.40	RANKING
8	3	43	-3.44	1.76	24.78	RANKING
9	1	47	-4.02	0.00	38.54	RANKING
9	2	59	-3.98	0.72	38.65	RANKING
9	3	60	-3.96	1.93	39.12	RANKING
9	4	4	-3.57	1.34	38.97	RANKING
10	1	16	-4.00	0.00	39.02	RANKING
10	2	23	-3.68	0.70	38.73	RANKING
11	1	30	-4.00	0.00	24.97	RANKING
11	2	31	-3.98	0.81	24.76	RANKING
12	1	40	-3.99	0.00	25.49	RANKING
12	2	45	-3.36	1.28	25.06	RANKING
12	3	2	-3.08	1.73	24.72	RANKING
13	1	3	-3.96	0.00	31.81	RANKING
14	1	15	-3.94	0.00	29.65	RANKING
15	1	56	-3.92	0.00	29.24	RANKING
15	2	5	-3.71	0.48	29.25	RANKING
16	1	37	-3.88	0.00	56.31	RANKING
17	1	52	-3.85	0.00	38.65	RANKING
18	1	48	-3.75	0.00	24.54	RANKING
19	1	35	-3.69	0.00	21.97	RANKING
19	2	17	-3.64	0.35	22.01	RANKING
19	3	58	-3.56	0.82	22.35	RANKING
20	1	8	-3.62	0.00	26.69	RANKING
21	1	34	-3.58	0.00	30.32	RANKING
21	2	41	-3.52	1.98	30.20	RANKING
22	1	14	-3.56	0.00	25.33	RANKING
23	1	6	-3.53	0.00	28.87	RANKING
24	1	1	-3.50	0.00	28.98	RANKING
25	1	20	-3.45	0.00	78.63	RANKING
26	1	21	-3.43	0.00	28.61	RANKING
26	2	19	-3.37	0.43	28.60	RANKING
26	3	50	-3.33	1.92	28.95	RANKING
27	1	7	-3.42	0.00	37.10	RANKING
28	1	27	-3.28	0.00	80.27	RANKING
28	2	49	-2.99	0.55	80.49	RANKING

29	1	36	-3.27	0.00	39.92	RANKING
30	1	11	-3.23	0.00	38.98	RANKING
31	1	38	-3.23	0.00	84.76	RANKING
32	1	10	-3.22	0.00	25.58	RANKING
33	1	32	-3.20	0.00	39.43	RANKING
34	1	33	-3.18	0.00	32.25	RANKING
35	1	18	-3.09	0.00	24.61	RANKING
36	1	29	-2.98	0.00	85.87	RANKING

1AV1: site-specific docking

```
LOWEST ENERGY DOCKED CONFORMATION
USER    Estimated Free Energy of Binding    =   -6.00 kcal/mol  [=(1)+(2)+(3)-(4)]
USER    Estimated Inhibition Constant, Ki   =   40.12 uM (micromolar)  [Temperature = 298.15 K]
USER
USER    (1) Final Intermolecular Energy     =   -7.79 kcal/mol
USER        vdW + Hbond + desolv Energy     =   -7.07 kcal/mol
USER        Electrostatic Energy            =   -0.72 kcal/mol
USER    (2) Final Total Internal Energy     =   -1.17 kcal/mol
USER    (3) Torsional Free Energy           =   +1.79 kcal/mol
USER    (4) Unbound System's Energy  [=(2)] =   -1.17 kcal/mol
```

Rank	Sub-Rank	Run	Binding Energy	Cluster RMSD	Reference RMSD	Grep Pattern
1	1	58	-6.00	0.00	31.23	RANKING
1	2	15	-5.96	0.90	30.99	RANKING
1	3	45	-5.91	0.74	31.05	RANKING
1	4	25	-5.87	1.13	31.22	RANKING
1	5	59	-5.86	1.12	31.20	RANKING
1	6	43	-5.86	0.75	31.51	RANKING
1	7	1	-5.78	0.82	31.04	RANKING
1	8	20	-5.68	0.80	31.15	RANKING
1	9	35	-5.40	0.64	31.48	RANKING
1	10	29	-5.16	1.75	31.74	RANKING
2	1	49	-5.96	0.00	32.26	RANKING
2	2	55	-5.94	0.12	32.27	RANKING
2	3	31	-5.93	0.21	32.30	RANKING
2	4	44	-5.92	0.11	32.31	RANKING
2	5	3	-5.91	0.28	32.23	RANKING
2	6	30	-5.90	0.16	32.31	RANKING
2	7	4	-5.88	0.26	32.27	RANKING
2	8	46	-5.87	0.21	32.29	RANKING
2	9	19	-5.87	0.38	32.31	RANKING
2	10	48	-5.86	0.21	32.38	RANKING
2	11	50	-5.86	0.23	32.34	RANKING
2	12	8	-5.85	0.34	32.36	RANKING
2	13	23	-5.84	0.31	32.36	RANKING
2	14	60	-5.77	1.95	31.73	RANKING
2	15	54	-5.68	1.06	32.83	RANKING
2	16	51	-5.68	1.05	32.82	RANKING
2	17	14	-5.65	1.03	32.87	RANKING
2	18	53	-5.62	0.82	32.60	RANKING
2	19	18	-5.58	0.81	32.60	RANKING
2	20	26	-5.57	1.02	32.84	RANKING
2	21	16	-5.31	1.07	32.55	RANKING
2	22	37	-5.27	1.00	32.86	RANKING
2	23	33	-5.10	1.13	32.91	RANKING
3	1	24	-5.48	0.00	32.25	RANKING
4	1	39	-5.36	0.00	33.60	RANKING
4	2	17	-5.32	1.06	33.97	RANKING
5	1	21	-5.29	0.00	32.06	RANKING
5	2	6	-5.28	0.15	32.07	RANKING
5	3	10	-5.28	0.17	32.06	RANKING
5	4	34	-5.27	0.07	32.08	RANKING
5	5	7	-5.27	0.17	32.06	RANKING
5	6	41	-5.27	0.17	32.05	RANKING
5	7	57	-5.26	0.18	32.07	RANKING
5	8	27	-5.26	0.18	32.03	RANKING
5	9	32	-5.26	0.17	32.07	RANKING
5	10	47	-5.26	0.15	32.06	RANKING

5	11	11	-5.24	0.19	32.09	RANKING
5	12	2	-5.24	0.20	32.07	RANKING
5	13	13	-5.24	0.16	32.06	RANKING
5	14	28	-5.24	0.10	32.11	RANKING
5	15	40	-5.22	0.14	32.10	RANKING
5	16	36	-5.19	0.20	32.10	RANKING
5	17	42	-5.16	0.25	32.12	RANKING
5	18	5	-5.14	0.25	32.14	RANKING
5	19	22	-5.14	0.27	32.14	RANKING
5	20	12	-5.12	0.27	32.11	RANKING
5	21	9	-5.11	1.14	32.40	RANKING
5	22	52	-5.07	1.14	32.38	RANKING
6	1	38	-5.12	0.00	32.55	RANKING
6	2	56	-5.11	0.06	32.55	RANKING

2N5E: blind docking

```
LOWEST ENERGY DOCKED CONFORMATION
USER    Estimated Free Energy of Binding    =   -4.58 kcal/mol  [=(1)+(2)+(3)-(4)]
USER    Estimated Inhibition Constant, Ki   =  437.48 uM (micromolar)  [Temperature = 298.15 K]
USER
USER    (1) Final Intermolecular Energy     =   -6.37 kcal/mol
USER        vdW + Hbond + desolv Energy     =   -6.22 kcal/mol
USER        Electrostatic Energy            =   -0.16 kcal/mol
USER    (2) Final Total Internal Energy     =   -1.27 kcal/mol
USER    (3) Torsional Free Energy           =   +1.79 kcal/mol
USER    (4) Unbound System's Energy  [=(2)] =   -1.27 kcal/mol
```

Rank	Sub-Rank	Run	Binding Energy	Cluster RMSD	Reference RMSD	Grep Pattern
1	1	11	-4.58	0.00	25.83	RANKING
1	2	31	-4.52	0.96	26.19	RANKING
1	3	34	-4.45	0.96	25.80	RANKING
2	1	39	-4.54	0.00	27.60	RANKING
2	2	48	-4.45	0.50	27.64	RANKING
2	3	38	-4.05	0.96	28.17	RANKING
2	4	52	-3.90	0.99	28.18	RANKING
3	1	54	-4.34	0.00	95.76	RANKING
4	1	8	-4.32	0.00	33.13	RANKING
4	2	49	-4.23	1.13	32.61	RANKING
4	3	25	-4.07	1.95	33.94	RANKING
4	4	50	-3.94	1.96	33.55	RANKING
4	5	27	-3.91	1.26	33.50	RANKING
4	6	35	-3.91	1.92	33.89	RANKING
4	7	26	-3.88	0.99	32.87	RANKING
5	1	29	-4.31	0.00	31.32	RANKING
5	2	56	-4.30	0.14	31.30	RANKING
5	3	33	-4.24	1.12	31.47	RANKING
5	4	4	-4.24	0.18	31.34	RANKING
5	5	17	-4.17	0.36	31.37	RANKING
5	6	13	-4.16	0.36	31.37	RANKING
5	7	19	-4.15	0.37	31.35	RANKING
5	8	5	-4.13	0.42	31.37	RANKING
5	9	24	-4.12	0.33	31.37	RANKING
5	10	22	-4.03	0.91	30.97	RANKING
5	11	45	-3.65	1.60	31.39	RANKING
6	1	53	-4.16	0.00	33.70	RANKING
6	2	47	-3.75	1.29	33.81	RANKING
6	3	58	-3.57	1.06	34.14	RANKING
7	1	23	-4.16	0.00	23.34	RANKING
8	1	30	-4.11	0.00	29.80	RANKING
9	1	32	-4.10	0.00	33.38	RANKING
10	1	43	-4.09	0.00	28.56	RANKING
11	1	1	-4.01	0.00	23.11	RANKING
11	2	18	-3.94	0.30	23.23	RANKING
12	1	21	-4.01	0.00	24.91	RANKING
13	1	60	-3.99	0.00	26.26	RANKING
14	1	10	-3.97	0.00	31.73	RANKING

15	1	59	-3.94	0.00	30.07	RANKING
16	1	6	-3.89	0.00	26.65	RANKING
17	1	12	-3.88	0.00	25.76	RANKING
18	1	40	-3.83	0.00	23.57	RANKING
18	2	51	-3.77	0.41	23.62	RANKING
19	1	2	-3.81	0.00	95.58	RANKING
20	1	20	-3.80	0.00	27.14	RANKING
21	1	41	-3.78	0.00	91.41	RANKING
21	2	57	-3.56	0.39	91.47	RANKING
22	1	14	-3.75	0.00	72.80	RANKING
23	1	3	-3.72	0.00	27.23	RANKING
24	1	44	-3.67	0.00	28.82	RANKING
25	1	7	-3.58	0.00	24.41	RANKING
26	1	37	-3.58	0.00	77.50	RANKING
27	1	9	-3.46	0.00	38.71	RANKING
28	1	55	-3.46	0.00	35.34	RANKING
29	1	28	-3.39	0.00	89.75	RANKING
30	1	36	-3.38	0.00	26.23	RANKING
31	1	16	-3.25	0.00	28.22	RANKING
32	1	46	-3.10	0.00	44.18	RANKING
33	1	42	-3.10	0.00	53.80	RANKING
34	1	15	-2.93	0.00	36.47	RANKING

2N5E: site-specific docking

```
LOWEST ENERGY DOCKED CONFORMATION
USER    Estimated Free Energy of Binding    =   -6.12 kcal/mol  [=(1)+(2)+(3)-(4)]
USER    Estimated Inhibition Constant, Ki   =   32.73 uM (micromolar)  [Temperature = 298.15 K]
USER
USER    (1) Final Intermolecular Energy     =   -7.91 kcal/mol
USER        vdW + Hbond + desolv Energy     =   -7.80 kcal/mol
USER        Electrostatic Energy            =   -0.11 kcal/mol
USER    (2) Final Total Internal Energy     =   -1.47 kcal/mol
USER    (3) Torsional Free Energy           =   +1.79 kcal/mol
USER    (4) Unbound System's Energy  [=(2)] =   -1.47 kcal/mol
```

Rank	Sub-Rank	Run	Binding Energy	Cluster RMSD	Reference RMSD	Grep Pattern
1	1	43	-6.12	0.00	25.11	RANKING
1	2	26	-6.09	0.49	25.29	RANKING
1	3	50	-6.09	0.85	25.43	RANKING
1	4	37	-6.08	0.90	25.40	RANKING
1	5	9	-6.07	0.16	25.18	RANKING
1	6	46	-6.06	0.84	25.42	RANKING
1	7	15	-6.05	0.47	25.24	RANKING
1	8	7	-6.05	1.64	25.72	RANKING
1	9	35	-6.04	0.86	25.45	RANKING
1	10	5	-6.04	0.30	25.23	RANKING
1	11	30	-6.03	0.62	25.36	RANKING
1	12	14	-5.97	0.87	25.43	RANKING
1	13	24	-5.95	0.87	25.48	RANKING
1	14	47	-5.88	1.96	25.89	RANKING
1	15	48	-5.88	0.43	25.24	RANKING
1	16	28	-5.76	1.35	25.84	RANKING
1	17	31	-5.60	1.48	25.86	RANKING
2	1	2	-5.90	0.00	26.19	RANKING
2	2	52	-5.89	0.99	26.48	RANKING
2	3	21	-5.88	1.55	25.73	RANKING
2	4	12	-5.86	1.61	25.65	RANKING
2	5	20	-5.86	1.58	25.84	RANKING
2	6	18	-5.85	1.54	25.74	RANKING
2	7	51	-5.82	1.58	25.64	RANKING
2	8	16	-5.81	1.54	25.82	RANKING
2	9	55	-5.81	1.57	25.74	RANKING
2	10	11	-5.81	0.63	26.17	RANKING
2	11	39	-5.80	0.81	26.45	RANKING
2	12	23	-5.80	0.79	26.30	RANKING
2	13	6	-5.80	1.68	25.70	RANKING

2	14	45	-5.80	0.76	26.33	RANKING
2	15	60	-5.80	1.50	25.91	RANKING
2	16	27	-5.80	1.56	25.67	RANKING
2	17	22	-5.79	0.79	26.40	RANKING
2	18	10	-5.79	1.59	25.71	RANKING
2	19	56	-5.77	0.76	26.29	RANKING
2	20	1	-5.75	1.59	25.68	RANKING
2	21	41	-5.72	0.92	26.53	RANKING
2	22	17	-5.70	1.12	25.87	RANKING
2	23	19	-5.69	0.80	26.33	RANKING
2	24	58	-5.67	1.39	25.95	RANKING
2	25	29	-5.65	1.30	25.76	RANKING
2	26	33	-5.61	1.04	25.91	RANKING
2	27	36	-5.61	0.81	26.32	RANKING
2	28	32	-5.58	0.77	26.32	RANKING
2	29	38	-5.55	0.74	26.37	RANKING
2	30	4	-5.51	1.29	25.68	RANKING
2	31	40	-5.30	1.56	26.38	RANKING
3	1	53	-5.90	0.00	26.05	RANKING
3	2	49	-5.82	0.32	26.12	RANKING
3	3	54	-5.78	0.59	26.06	RANKING
4	1	3	-5.58	0.00	25.79	RANKING
4	2	59	-5.48	0.48	25.90	RANKING
5	1	13	-5.44	0.00	26.26	RANKING
6	1	25	-5.44	0.00	26.92	RANKING
6	2	44	-5.43	0.47	26.69	RANKING
6	3	34	-5.40	0.56	26.62	RANKING
6	4	57	-5.34	0.64	26.63	RANKING
7	1	8	-5.11	0.00	25.15	RANKING
8	1	42	-4.95	0.00	23.30	RANKING

3R2P: blind docking

```
LOWEST ENERGY DOCKED CONFORMATION
USER    Estimated Free Energy of Binding    =   -5.80 kcal/mol  [=(1)+(2)+(3)-(4)]
USER    Estimated Inhibition Constant, Ki   =   55.95 uM (micromolar)  [Temperature = 298.15 K]
USER
USER    (1) Final Intermolecular Energy     =   -7.59 kcal/mol
USER        vdW + Hbond + desolv Energy     =   -7.00 kcal/mol
USER        Electrostatic Energy            =   -0.59 kcal/mol
USER    (2) Final Total Internal Energy     =   -1.48 kcal/mol
USER    (3) Torsional Free Energy           =   +1.79 kcal/mol
USER    (4) Unbound System's Energy  [=(2)] =   -1.48 kcal/mol
```

Rank	Sub-Rank	Run	Binding Energy	Cluster RMSD	Reference RMSD	Grep Pattern
1	1	18	-5.80	0.00	28.29	RANKING
1	2	31	-5.78	0.52	28.08	RANKING
1	3	59	-5.26	0.87	28.19	RANKING
1	4	12	-5.16	0.90	28.46	RANKING
1	5	51	-5.05	0.83	28.83	RANKING
1	6	34	-4.97	0.94	28.27	RANKING
1	7	10	-4.93	1.27	29.09	RANKING
2	1	46	-5.12	0.00	54.23	RANKING
2	2	45	-4.15	1.01	54.54	RANKING
3	1	7	-5.03	0.00	31.80	RANKING
3	2	11	-4.86	0.98	32.41	RANKING
3	3	48	-4.84	0.79	32.32	RANKING
3	4	21	-4.84	0.26	31.89	RANKING
3	5	54	-4.48	1.80	32.20	RANKING
3	6	9	-4.40	1.79	32.07	RANKING
3	7	47	-4.26	1.82	32.03	RANKING
4	1	25	-4.97	0.00	46.29	RANKING
4	2	38	-4.90	0.39	46.34	RANKING
4	3	42	-4.87	1.33	46.87	RANKING
4	4	55	-4.42	1.96	47.24	RANKING
4	5	20	-4.04	1.74	47.41	RANKING
5	1	29	-4.97	0.00	28.54	RANKING

5	2	49	-4.79	1.99	28.19	RANKING
5	3	53	-4.68	1.95	28.08	RANKING
5	4	2	-4.42	1.92	28.51	RANKING
6	1	22	-4.89	0.00	27.96	RANKING
6	2	27	-4.78	1.09	28.23	RANKING
7	1	52	-4.84	0.00	39.41	RANKING
8	1	58	-4.72	0.00	26.95	RANKING
9	1	8	-4.69	0.00	29.56	RANKING
9	2	19	-4.64	0.25	29.43	RANKING
9	3	30	-4.52	1.88	30.47	RANKING
9	4	13	-4.47	1.86	30.47	RANKING
9	5	37	-4.25	1.76	30.61	RANKING
9	6	41	-4.24	1.95	30.47	RANKING
10	1	40	-4.48	0.00	20.69	RANKING
11	1	39	-4.38	0.00	54.54	RANKING
12	1	57	-4.32	0.00	28.85	RANKING
13	1	26	-4.28	0.00	18.06	RANKING
14	1	14	-4.28	0.00	31.71	RANKING
15	1	6	-4.25	0.00	30.32	RANKING
15	2	32	-4.09	0.48	30.03	RANKING
16	1	23	-4.25	0.00	30.13	RANKING
17	1	28	-4.22	0.00	34.80	RANKING
18	1	36	-4.18	0.00	31.42	RANKING
18	2	60	-4.10	0.70	31.56	RANKING
19	1	24	-4.16	0.00	30.83	RANKING
20	1	43	-4.14	0.00	48.75	RANKING
21	1	35	-4.12	0.00	27.43	RANKING
22	1	33	-4.10	0.00	46.40	RANKING
23	1	44	-4.04	0.00	30.83	RANKING
23	2	4	-3.83	1.22	30.93	RANKING
24	1	56	-4.04	0.00	16.82	RANKING
25	1	16	-3.98	0.00	36.02	RANKING
26	1	50	-3.95	0.00	32.12	RANKING
27	1	15	-3.80	0.00	20.86	RANKING
28	1	5	-3.70	0.00	55.55	RANKING
28	2	17	-3.62	1.64	55.56	RANKING
29	1	3	-3.70	0.00	51.35	RANKING
30	1	1	-3.44	0.00	49.36	RANKING

3R2P: site-specific docking

```
LOWEST ENERGY DOCKED CONFORMATION
USER    Estimated Free Energy of Binding    =   -7.54 kcal/mol  [=(1)+(2)+(3)-(4)]
USER    Estimated Inhibition Constant, Ki   =    2.99 uM (micromolar)  [Temperature = 298.15 K]
USER
USER    (1) Final Intermolecular Energy     =   -9.33 kcal/mol
USER        vdW + Hbond + desolv Energy     =   -8.85 kcal/mol
USER        Electrostatic Energy            =   -0.48 kcal/mol
USER    (2) Final Total Internal Energy     =   -1.37 kcal/mol
USER    (3) Torsional Free Energy           =   +1.79 kcal/mol
USER    (4) Unbound System's Energy  [=(2)] =   -1.37 kcal/mol
```

Rank	Sub-Rank	Run	Binding Energy	Cluster RMSD	Reference RMSD	Grep Pattern
1	1	28	-7.54	0.00	28.15	RANKING
1	2	39	-7.52	0.17	28.13	RANKING
1	3	60	-7.52	0.46	28.31	RANKING
1	4	7	-7.52	0.23	28.20	RANKING
1	5	2	-7.52	0.22	28.24	RANKING
1	6	23	-7.51	0.47	28.38	RANKING
1	7	13	-7.50	0.21	28.18	RANKING
1	8	51	-7.50	0.48	28.16	RANKING
1	9	33	-7.49	0.23	28.19	RANKING
1	10	9	-7.48	0.40	28.11	RANKING
1	11	52	-7.48	0.53	28.43	RANKING
1	12	41	-7.48	0.52	28.40	RANKING
1	13	55	-7.47	0.21	28.12	RANKING
1	14	24	-7.44	0.61	28.38	RANKING

1	15	38	-7.43	0.41	28.14	RANKING
1	16	42	-7.41	0.74	28.00	RANKING
1	17	22	-7.41	0.83	27.98	RANKING
1	18	57	-7.41	0.73	28.02	RANKING
1	19	37	-7.39	0.65	28.52	RANKING
1	20	54	-7.38	0.57	28.05	RANKING
1	21	20	-7.36	0.85	28.08	RANKING
1	22	17	-7.36	0.89	28.09	RANKING
1	23	53	-7.36	0.55	28.07	RANKING
1	24	5	-7.35	0.87	28.03	RANKING
1	25	56	-7.34	0.45	28.07	RANKING
1	26	31	-7.33	0.63	28.03	RANKING
1	27	49	-7.33	0.30	28.13	RANKING
1	28	40	-7.31	0.47	28.11	RANKING
1	29	46	-7.27	0.29	28.18	RANKING
1	30	29	-7.24	0.62	28.11	RANKING
1	31	3	-7.20	1.01	27.89	RANKING
1	32	48	-7.18	0.41	28.11	RANKING
1	33	18	-7.15	0.42	28.17	RANKING
1	34	6	-7.15	1.17	28.15	RANKING
1	35	4	-7.14	0.44	28.12	RANKING
1	36	35	-7.13	1.06	27.96	RANKING
1	37	58	-7.12	0.70	28.62	RANKING
1	38	59	-7.02	0.54	28.14	RANKING
1	39	21	-6.87	1.02	28.25	RANKING
1	40	50	-6.87	0.99	28.28	RANKING
1	41	36	-6.87	1.00	28.29	RANKING
1	42	16	-6.86	1.03	28.26	RANKING
1	43	43	-6.85	0.72	28.06	RANKING
1	44	34	-6.84	1.07	28.25	RANKING
1	45	12	-6.84	0.97	28.21	RANKING
1	46	27	-6.84	1.05	28.28	RANKING
1	47	19	-6.82	1.00	28.30	RANKING
1	48	10	-6.82	1.03	28.26	RANKING
1	49	15	-6.80	0.93	28.27	RANKING
1	50	8	-6.78	0.98	28.22	RANKING
1	51	11	-6.70	0.98	28.32	RANKING
1	52	32	-6.67	1.08	28.30	RANKING
1	53	30	-6.60	1.02	28.36	RANKING
1	54	14	-6.60	1.05	28.31	RANKING
1	55	26	-6.60	1.13	28.52	RANKING
1	56	25	-6.37	1.76	27.71	RANKING
1	57	1	-6.28	1.34	29.20	RANKING
1	58	47	-6.17	1.40	29.00	RANKING
1	59	44	-6.16	1.79	27.76	RANKING
2	1	45	-5.48	0.00	23.05	RANKING

III. Surface plasmon resonance raw data

Values of kinetic constants and binding affinities for the interactions of fentanyl and prasugrel metabolite with human plasma proteins obtained from the individual experiments.

Ligand	Analyte	k_a [1/Ms]	k_d [1/s]	K_A [1/M]	K_D [M]	χ^2
HSA	Fentanyl	1.10×10^{3}	4.45×10^{-3}	2.46×10^{5}	4.06×10^{-6}	0.406
HSA	Fentanyl	7.88×10^{2}	2.47×10^{-2}	3.19×10^{4}	3.13×10^{-5}	0.605
HSA	Fentanyl	1.24×10^{4}	3.15×10^{-2}	3.93×10^{5}	2.55×10^{-6}	0.386
AGP	Fentanyl	5.94×10^{0}	3.81×10^{-3}	1.56×10^{3}	6.41×10^{-4}	0.259
AGP	Fentanyl	6.70×10^{2}	8.30×10^{-7}	8.08×10^{8}	1.24×10^{-9}	0.132
AGP	Fentanyl	1.09×10^{3}	8.85×10^{-3}	1.23×10^{5}	8.13×10^{-6}	0.179
ApoA	Fentanyl	3.67×10^{2}	8.54×10^{-7}	4.29×10^{8}	2.33×10^{-9}	0.918
ApoA	Fentanyl	3.32×10^{2}	2.24×10^{-7}	1.48×10^{9}	6.74×10^{-10}	0.806
ApoA	Fentanyl	3.27×10^{2}	2.46×10^{-6}	1.33×10^{8}	7.50×10^{-9}	0.860
ApoB	Fentanyl	1.44×10^{2}	2.30×10^{-2}	6.24×10^{3}	1.60×10^{-4}	0.865
ApoB	Fentanyl	1.43×10^{2}	2.46×10^{-2}	5.82×10^{3}	1.72×10^{-4}	0.653
ApoB	Fentanyl	1.16×10^{2}	2.79×10^{-2}	4.16×10^{3}	2.40×10^{-4}	1.320
ApoA	Prasugrel metabolite	4.64×10^{2}	1.67×10^{-2}	2.78×10^{4}	3.60×10^{-5}	0.306
ApoA	Prasugrel metabolite	3.12×10^{3}	8.98×10^{-8}	3.48×10^{10}	2.88×10^{-11}	0.637
ApoA	Prasugrel metabolite	2.69×10^{3}	7.78×10^{-6}	3.46×10^{8}	2.89×10^{-9}	0.347
ApoB	Prasugrel metabolite	2.44×10^{3}	5.74×10^{-6}	4.25×10^{8}	2.35×10^{-9}	1.140
ApoB	Prasugrel metabolite	1.67×10^{3}	5.08×10^{-6}	3.30×10^{8}	3.03×10^{-9}	0.551
ApoB	Prasugrel metabolite	3.61×10^{3}	4.06×10^{-6}	8.88×10^{8}	1.13×10^{-9}	0.843
