# Supplementary material for: Interactions of fentanyl with blood platelets and plasma proteins: platelet sensitivity to prasugrel metabolite is not affected by fentanyl under in vitro conditions
Source: Pharmacol Rep. 2023 Jan 17;75(2):423–41. doi: 10.1007/s43440-023-00447-7 (PMC10060303; doi:10.1007/s43440-023-00447-7)
Supplement: Supplementary file 2 — Supplementary file2 (PDF 209 KB) [file 43440_2023_447_MOESM2_ESM.pdf]

Interactions of fentanyl with blood platelets and plasma proteins: platelet sensitivity to prasugrel metabolite is not affected by fentanyl under in vitro conditions

SUPPLEMENTARY FIGURES

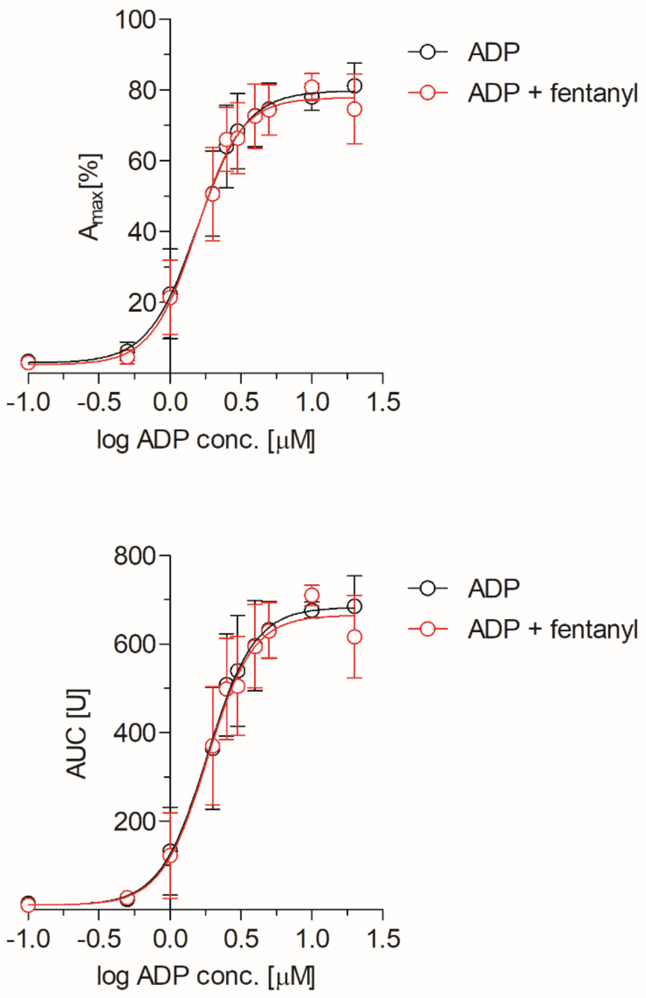

Supplementary Figure S1. The effect of fentanyl on ADP-induced platelet aggregation. The amplitude (Amax) and AUC values of platelet aggregation were measured in platelet-rich plasma by LTA, in response to 0.1–20 µM ADP. Results are shown as mean ± SEM (n = 5) for ADP alone and ADP + fentanyl (2 µg/ml). The EC_{50} values calculated from dose-response plots were 1.5 µM for ADP and 1.8 µM for ADP + fentanyl.

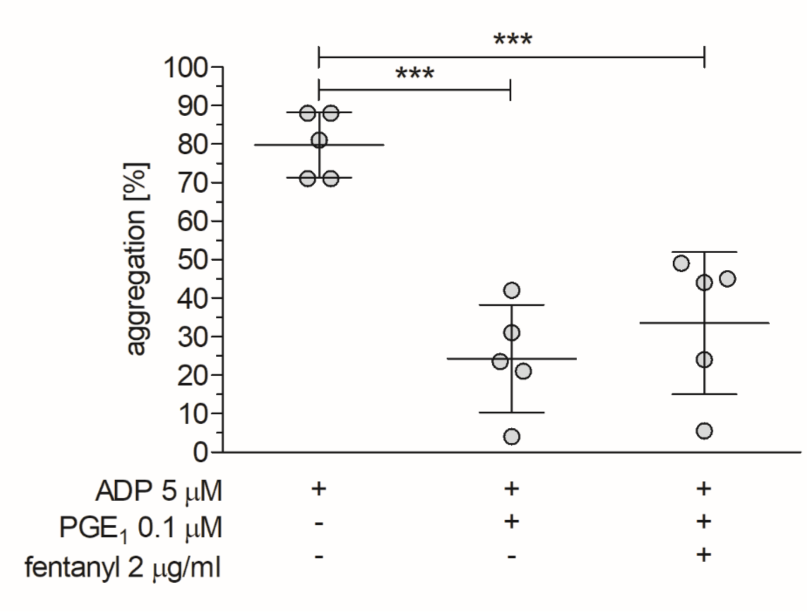

Supplementary Figure S2. The effect of fentanyl on PGE_1-mediated inhibition of platelet aggregation. Platelet aggregation was measured in platelet-rich plasma by LTA, in response to 5 μM ADP. The antagonism between fentanyl and PGE_1 was assessed after one-minute preincubation of PRP with PGE_1 in the presence of fentanyl. Results are shown as mean ± SD (n = 5). Statistically significant differences were estimated by repeated measures ANOVA followed by post hoc Tukey's multiple comparisons test. ***$p < 0.001$ vs. control.
